# Integrating Constitutive Gene Expression and Chemoactivity: Mining the NCI60 Anticancer Screen

**DOI:** 10.1371/journal.pone.0044631

**Published:** 2012-10-02

**Authors:** David G. Covell

**Affiliations:** Developmental Therapeutics Program, Frederick National Laboratory, National Institutes of Health, Frederick, Maryland, United States of America; University of Illinois at Chicago, United States of America

## Abstract

Studies into the genetic origins of tumor cell chemoactivity pose significant challenges to bioinformatic mining efforts. Connections between measures of gene expression and chemoactivity have the potential to identify clinical biomarkers of compound response, cellular pathways important to efficacy and potential toxicities; all vital to anticancer drug development. An investigation has been conducted that jointly explores tumor-cell constitutive NCI60 gene expression profiles and small-molecule NCI60 growth inhibition chemoactivity profiles, viewed from novel applications of self-organizing maps (SOMs) and pathway-centric analyses of gene expressions, to identify subsets of over- and under-expressed pathway genes that discriminate chemo-sensitive and chemo-insensitive tumor cell types. Linear Discriminant Analysis (LDA) is used to quantify the accuracy of discriminating genes to predict tumor cell chemoactivity. LDA results find 15% higher prediction accuracies, using ∼30% fewer genes, for pathway-derived discriminating genes when compared to genes derived using conventional gene expression-chemoactivity correlations. The proposed pathway-centric data mining procedure was used to derive discriminating genes for ten well-known compounds. Discriminating genes were further evaluated using gene set enrichment analysis (GSEA) to reveal a cellular genetic landscape, comprised of small numbers of key over and under expressed on- and off-target pathway genes, as important for a compound’s tumor cell chemoactivity. Literature-based validations are provided as support for chemo-important pathways derived from this procedure. Qualitatively similar results are found when using gene expression measurements derived from different microarray platforms. The data used in this analysis is available at http://pubchem.ncbi.nlm.nih.gov/and http://www.ncbi.nlm.nih.gov/projects/geo (GPL96, GSE32474).

## Introduction

Anticancer drug discovery continues to be a task of paramount importance [1] and enormous scientific challenge [2]. Faced with clinical findings that the expected promise of on-target therapies remains only partially fulfilled [3], strategies for improvement have motivated the emergence of considerable publicly accessible, information-rich data [4], [5] and data mining strategies [6]. Clinical and basic science findings also suggest that therapeutic efficacy may arise from multiple factors [7] such as gene expression levels, mutation status and single nucleotide polymorphisms, each of which may potentially involve numerous, on-target and off-target molecules [8]. While the importance of these diverse factors on compound efficacy continues to be actively pursued [9], the challenge of linking measures of on- and off-target gene expressions to small molecule screening chemoactivity continues to hold promise for identifying cellular pathways important to efficacy [10], clinical biomarkers of compound response [11], [12] and potential toxicities [13], [14]; all vital to anticancer drug discovery.

Chemoactivity studies have fostered publically available screening databases such as PubChem [4], [13], [14] and ToxRefDB [15], [16]. The PubChem database includes results from the National Cancer Institute’s *in vitro* tumor cell screen (referred to as the NCI60 [17]) for potential anticancer agents. Historically, NCI60 screening measurements of growth inhibition (referred to as GI50 measures) have yielded valuable insights into a compound’s cellular mechanism of action [18]–[20], as well as inspiring the development and validation of computational and statistical data mining tools [21]–[24]. Cell-based assays extend on-target, molecular screening results by also including roles for off-target effectors in a cellular response. Oftentimes cellular screening efforts are accompanied by baseline gene expression measurements. Prior correlative studies of chemoactivity and gene expressions have, however, found relatively few meaningful correlations [21], [25], and inspired the proposal of more elaborate computational means of identifying compound-target associations [26]. A specific limitation of direct correlative means to identify a putative target appears in Nakatsu *et al.* [18] where a panel of 45 human tumor cell lines was used to identify genes important for the chemoactivity of 53 anticancer drugs. While gene profile clustering was able to identify compounds sharing a common putative mode of action, explicit correlation of chemoactivity with gene expressions did not identify putative targets. Their study found that slightly more than two dozen gene expressions were positively correlated with cellular growth inhibition from the camptothecin (CPT) analog, SN-38. None of these genes included the CPT target topoisomerase I. This observation was repeated for the tubulin targeting agent paclitaxel, where its set of 22 chemosensitive correlated gene expressions excluded members of the tubulin family. These results serve to underscore the potential limitations of using chemoactivity-gene expression correlations, directly, to identify chemo-important genes.

A promising complement to studying relationships between cellular chemoactivity and gene expressions utilizes annotations of genes into pathways. Pathways, while often incompletely established, offer an opportunity to explore cassettes of genes within annotated pathways for the influence of their gene expressions on biological responses. Currently large numbers of pathway annotations, comprised, typically, of tens to hundreds of genes, are available for gene-based studies of public cellular chemoactivity databases. Supporting these pathway annotations are software tools for analyzing pathway genes (IPA(Ingenuity^R^ Systems www.ingenuity.com), GSEA [27], [28] and DAVID [29]). At first glance, *in silico* mining strategies for associating pathways and their component gene expressions to cellular chemoactivity appear to represent an additional complication to the already challenging issues resulting from the roles of on-target and off-target effectors. Alternatively, pathway-centric approaches have been used previously in conjunction with cellular screening data to explore correlations between gene products and pathways for purposes of identifying interesting cancer targets [23]. Their pathway-centric approach found a general tendency for gene expression to become less coherent in tumor versus normal tissues, especially for signaling pathways, with pathways containing known cancer genes (i.e., “cancer pathways”) amongst the least coherent pathways; a result not apparent from direct examinations of individual gene expressions [23], [30]. These results suggest that pathway-centric data mining strategies may provide a new alternative to that of exploring direct associations between pathway gene expressions and cellular chemoactivity.

The analysis presented here represents a novel data mining strategy, developed from a pathway-centric viewpoint, to examine relationships between cellular gene expressions and cellular chemoactivity. The results of applying this method to the NCI60 databases will establish the existence of complex genetic landscapes, comprised of on- and off- target pathway genes as important to cellular chemoactivity.

## Methods

A previously published method [23], [30], which uses conventional correlative chemoactivity-gene expression measures, has been modified to determine compound specific pathway scores (H-scores as described below) for a small set of test compounds. The best H-scores for each test compound identify important pathways and ‘discriminating’ genes from these pathways can be used for further analysis. This pathway-centric approach tends to favor instances where many pathway genes are coherently either over or under expressed, versus the conventional method that typically explores only the strongest correlations between gene expressions and cellular chemoactivity.

The analytic workflow begins by establishing a globally defined reference database for chemoactivity (referred to hereafter as the SOM GI50 reference database), followed by generation of pathway scores (H-scores) assigned to this reference database. The SOM GI50 reference database and its associated assignment of pathway H-scores provides a global perspective for viewing all available chemoactivity to pathway gene associations, and for selecting gene subsets that discriminate chemo-sensitive from chemo-insensitive tumor cell responses. As will be shown in the RESULTS, the conventional method of selecting only the most extreme pairwise gene expression-chemoactivity correlations typically yields discriminating genes different from this pathway-centric approach.

A summary of the analytic work flow involves:

- Creation of a reference database of NCI60 chemoactivity measures for screened small molecules
- Creation of a reference database of NCI60 gene expression profiles
- Assignment of pathway scores (H-scores) to the NCI60 chemoactivity reference database
- Selection of discriminating genes for each test compound
- Using Gene Set Enrichment Analysis (GSEA) of discriminating genes to provide statistical measures of pathway importance.
- Providing literature-based support for GSEA pathways, their associated gene expressions and chemoactivity for each test compound. These associations will be referred to as ‘pathway-gene chemoactivity’ associations.

The following sections will provide details related to this analytic workflow.

### Creation of a Reference Database of NCI60 Chemoactivity Measures for Screened Small Molecules

NCI60 GI50 values are used to define *in vitro* tumor cell chemoactivity for compounds screened by the Developmental Therapeutics Program (DTP). The complete GI50 data set is available in the PubChem repository [4]. Data filtering selects only GI50 profiles where more than 40 tumor cell lines reported values and excludes profiles with a coefficient of variation of less than 0.05 (i.e. minimal differential sensitivity, usually due to a compound’s insensitivity or pan cytotoxicity). GI50 records for over 60 k compounds remained after this filtering step. These GI50 records were normalized, first across tumor cell type, to remove any systematic biases due to individual tumor cell sensitivity, then within each record, to generate a GI50 z-score for each compound. These filtered and normalized records define a compound’s NCI60 GI50 profile. Each of these chemoactivity profiles can be used for correlative studies against gene expressions. Rather than develop a strategy utilizing all ∼60 k chemoactivity profiles, SOMs (Self-Organizing Maps [31]) were used to organize the filtered GI50 profiles into 1998 clusters (SOM map dimensions of 54X37). A more detailed description of SOMs applied to the NCI60 data can be found in Rabow *et al.* [24]. Noteworthy benefits of using SOM’s include it’s a) data reduction feature (60 k down to 2 k chemoactivity profiles, b) nonhierarchical clustering methodology, c) ability to analyze noisy data often with missing values and d) convenience for displaying clustering results (www.spheroid.ncifcrf.gov). In the calculations to follow, each SOM cluster will be characterized by a GI50 profile that most represents the growth inhibition for its cluster members. Each SOM cluster’s representative profile (also called a codebook vector in SOM nomenclature) will be referred to throughout the text as the SOM GI50 profile. Gene expressions correlations will be referenced to each SOM GI50 profile. Instances where raw GI50 measures are used for gene correlations will be clearly identified.

### Creation of a Reference Database of NCI60 Gene Expression Profiles

Constitutive(baseline) gene expressions for untreated NCI60 tumor cells were downloaded as GEO GPL96 [32]. These data were generated using the Affymetrix U133A chip. Earlier NCI60 expression data exists using the U95A Affymetrix chip. An average correlation coefficient of 0.82+/−0.21 was found when comparing NCI60 constitutive gene expressions between the U133A and U95A data sets. The U133A data set will be used for this analysis because it reported more expression values than the U95A data set and is in reasonable agreement with the U95A expression profiles. The U133A gene expression dataset consists of 22,282 probes, with 16,784 unique HUGO identifiers, that were reduced to 2,475 genes having a coefficient of variation greater than 0.05 and sharing at least one other gene in a curated pathway.

The results derived from the U133A gene expressions will be compared with those obtained from the recently released NCI60 baseline expressions measured using the U133Plus platform (GSE32474). This dataset consists of 54,675 probes, which represent 21,049 unique HUGO identifiers. Selection of gene expressions with a coefficient of variation greater than 0.05 reduced this set to 5,761 genes, with 65% (n = 3,721) of these genes appearing at least jointly in these curated pathways. Pathway gene-chemoactivity associations determined from the U133Plus gene expressions will be compared to those obtained when using the U133A gene expressions. The results will show that gene expressions from both platforms generally yield qualitatively similar results, while also revealing additional details about associations between gene expressions and chemoactivity that were not indicated with the U133A gene expressions. The analysis will report mainly on the results derived from the U133A gene expressions. Each test compound’s descriptive narrative will, however, include brief summaries and comparisons of the U133A and U133Plus-derived results.

### Assignment of Pathway Scores (H-scores) to the NCI60 Chemoactivity Reference Database

**Figure 1:**
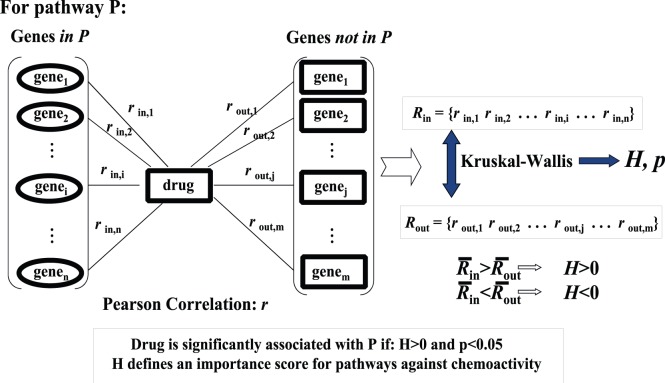
Schematic for calculating H-scores. For each pathway, P, select genes in P (ovals at left) and not in P (squares in middle). Calculate a Pearson correlation coefficient between all gene expressions and each SOM GI50 profile (designated as ‘drug’ in the central square). Use a Kruskal-Wallis(K-W) statistic to determine if the rankings of the pathway genes (ovals at left) versus non-pathway genes (squares in the middle) are significantly skewed towards extreme values. The H-score for each pathway is derived from the K-W statistic. Cases where the average of correlations associated with pathway genes is greater than the average of correlations for non-pathway genes characterize positive H-scores. Positive and significant (p<0.05) H-scores reflect pathways with coordinated (as opposed to random) gene expression-chemoactivity correlations. H-scores for each pathway (n = 2160) are determined for all SOM GI50 profiles (n = 1998).

The procedure to assign pathway scores uses a modification to a previously published pathway-centric approach [23], [30], [33]. The details of this process are: First; NCI60 constitutive genes are assigned to a pathway using GO, KEGG or Biocarta definitions. Only pathways having at least two NCI60 gene expression profiles were retained; yielding a total of 2160 pathways. Second; Assign pathway scores (H-scores) to each reference SOM GI50 node. This process consists of four steps; a) Generate correlations for all gene expression profiles to all SOM GI50 profiles. This step yields 1998×2547 correlations. b) Rank order the correlation values (n = 2547) for each GI50 SOM profile. c) For all pathways and each GI50 SOM profile identify the ranking of genes contained and excluded from a pathway. d) Apply a Kruskal-Wallis rank sum procedure to calculate a statistic (referred to as an H-score) as a measure of the non-random ranking of within versus excluded pathway genes. A schematic of the steps involved in calculating H-scores appears below in **Figure 1**. A more detailed description of calculating H-scores can be found in the Data and Methods section of Huang *et al.* [23]. The java code for H-score calculations and the output files are available upon request.

The underlying distribution of correlation values for gene expressions referenced to the test compound’s SOM GI50 profile is normally distributed within the 5 and 95 percentile range (i.e. linearity in a normal distribution plot, normplot in MATLAB, across the p> = 0.05 to p< = 0.95 range). In addition, the H-scores derived from these correlations are also normally distributed within the 5 to 95 percentile range. The highest fitness scores correspond to cases where the pathway gene expressions are concordantly strongly correlated with the reference SOM GI50 profile. These pathway fitness scores and their most correlated pathway genes occupy the tails of their respective distributions. Correlation values for non-pathway genes are scattered throughout the distribution of all correlations. The rank sum Kruskal-Wallis statistic is well-suited to identify pathways with gene expression correlations that are not randomly scattered throughout the overall distribution.

**Figure 2:**
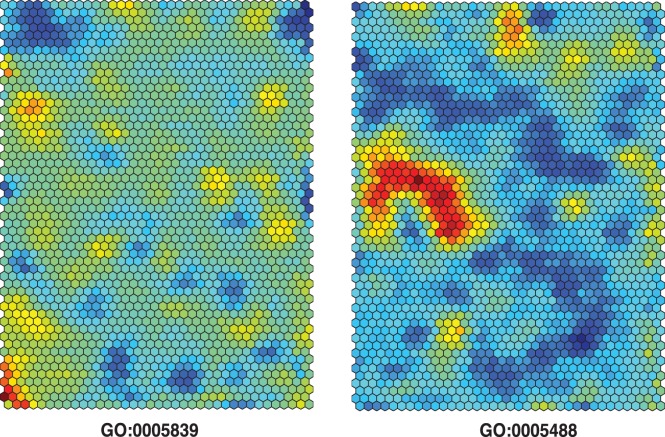
SOM GI50 projection of H-scores for the GO:proteasome pathway (GO:0005839, left panel) and the GO:binding pathway (GO:0005488, right panel). The SOM GI50 is represented as a 2 dimensional map of 54 rows by 37 columns, corresponding to 1998 clusters. Pathway H-scores are projected spectrally on the SOM GI50 (red: best H-score, blue: worst H-score). The lower left region of the leftmost GI50 SOM has the cluster containing camptothecin (CPT). This corresponds to the node with the highest H-score for the GO:proteasome pathway. The GO:binding pathway consists of genes associated primarily with organic acid transport, particularly into the mitochondrion. The majority of NCI60 screened compounds associated with the GO:0005488 SOM GI50 nodes having the highest H-scores contain multiple carboxylate groups.

Pathway H-scores can be projected on to the GI50 SOM for inspecting regions of possible pathway-chemoactivity associations. The examples in **Figure 2** project H-scores for the GO:Proteasome pathway (GO:0005839) and the GO:binding pathway (GO:0005488). The GO:proteasome pathway finds the best H-scores to be confined to the lower left SOM region, a region which includes the chemoactivity profile for camptothecin (CPT). The best H-scores for the GO:binding pathway appear as a downward facing horseshoe at the middle left region of the SOM. Each of these SOM regions includes screened compounds that would be hypothesized to derive their chemoactivity from roles in each respective pathway. It should be emphasized that the pathway H-scores can also be derived for each of the ∼60 k NCI60 chemoactivity profiles; rather than using the SOM GI50 profiles of the cluster containing each compound. The results will find that the topmost H-scores derived from either procedure yield similar sets of chemo-important pathways.

H-scores provide measures of importance for each pathway on a test compound’s chemoactivity. Since pathway H-scores are based on correlations between chemoactivity and gene expression, variations in pathway scores will depend on the choice of chemoactivity profile. The options available here include the SOM GI50 profile for the cluster containing the test compound, or the conventional raw GI50 profile of the test compound. To explore the overlap between H-scores derived from each choice of chemoactivity profile, a simulation was conducted consisting of randomly selecting 100 SOM GI50 nodes, and from each node randomly selecting 20 raw compound chemoactivity profiles. For each simulation, the topmost H-scores were determined using each of these 20 raw chemoactivity profiles and compared to the topmost H-scores obtained using the selected node’s GI50 SOM profile. These results find a mean fractional overlap between topmost scoring pathways derived from individual or SOM-based chemoactivity of 0.73 with a standard deviation of 0.15. This relatively high concordance of chemo-important pathways supports either choice of chemoactivity profile. While this choice may depend on a variety of user-defined reasons, selecting the SOM GI50 chemoactivity profile offers a number of advantages. Global surveys are more easily completed when using the SOM-based data reduction and organization features. Furthermore, each SOM GI50 chemoactivity profile represents its cluster member’s average cellular response, thus avoiding difficult questions about whether subtle differences tumor cell response arise from experimental noise or real biological differences. Finding nearly ¾ of the chemo-important pathways in common, when derived from either measure of chemoactivity, suggests that the group average response captures much of the cellular response shared by its cluster members. The robustness of finding many common pathways may also reflect the use of many correlation values when calculating each H-score, which tends to average out subtle differences between cellular responses from compounds with similar chemoactivity profiles. Based on this result, and the earlier cited advantages of using SOMs for data clustering, the pathway analysis to follow will be based on using SOM GI50 chemoactivity profiles when calculating H-scores.

### Selection of Discriminating Genes

Discriminating genes are derived for each test compound. The goal is to identify a minimal set of gene expression profiles that separate chemo-sensitive from chemo-insensitive tumor cells. The process begins by collecting all H-scores referenced to the GI50 SOM node containing each test compound. Gene expression profiles from pathways within the topmost 10^th^ percentile of H-scores are collected and filtered to include only gene expressions significantly (p<0.05) correlated with the test compound’s cellular chemoactivity. Selection of discriminating genes involves trimming this pathway-derived starting set of genes into four response classes:

over expressed/insensitive under expressed/sensitive

under expressed/insensitive over expressed/sensitive

**Figure 3:**
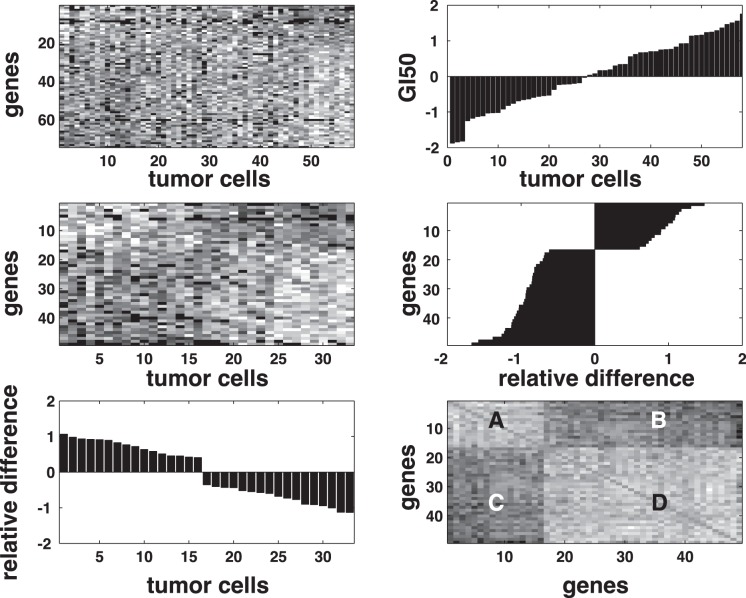
Illustration of the steps for trimming datasets. Upper left panel: row ordered (gene expressions) and column ordered (tumor cells) gene set derived from the topmost 10^th^ percentile of H-scores for this test example. Upper right panel: ordered chemoactivity profile (insensitive and sensitive tumor cells appear as + and - responses, respectively. Middle left panel: Ordered gene expressions for the trimmed set of discriminating genes. Middle right panel: Group averaged differential in gene expressions for chemo-sensitive and chemo-insensitive tumor cells. Lower left panel: Group averaged differential in gene expressions for over and under expressed discriminating genes. Lower right panel: Pearson correlation values (light:positive, dark:negative) for discriminating gene expressions. Letters correspond to response classes; A: over expressed/insensitive, B: under expressed/sensitive, C: under expressed/insensitive and D: over expressed/sensitive.

Data trimming eliminates pathway genes with weak correlations to chemoactivity and tumor cells that lack strongly differential chemoactivity responses. A two-step iterative application of a Student’s t-test is used for data trimming. **Figure 3** provides a sample illustration of this trimming process. The initial pathway-derived, filtered (p<0.05), dataset is ordered from left to right according to insensitive or sensitive chemoactivity, and top to bottom according to correlation strength between gene expression and chemoactivity (top most negatively correlated, bottom most positively correlated). The top left panel displays this ordered data, while the top right panel displays the ordered SOM GI50 chemoactivity profile, where negative and positive chemoactivity define insensitive and sensitive tumor cells, respectively. In this test case there were 68 pathway genes in the topmost pathway H-scores, each significantly correlated with the reference chemoactivity profile. For each iteration step a Student’s t-test is performed between the gene expressions in the sensitive versus insensitive tumor cells. Only genes with a significant (p<0.05) difference in expression between these tumor cell groups are accepted. These accepted genes are then subjected to a Student’s t-test based on groupings determined by over or under gene expression within the sensitive and insensitive tumor cells. Here tumor cells lacking a significant difference between over and under expressed genes are excluded from the next iteration. Iterations of this trimming process are terminated when all members of each groups are statistically separable in the row (gene expression) and column (cellular chemoactivity) dimensions. In this example, the initial gene set is reduced to 48 genes and 33 tumor cells (middle left panel). The middle right and lower left panels display, respectively, the differential in group values for chemoactivity (sensitive/insensitive) and gene expression (over/under). The lower right panel displays the gene-gene correlations for the final set of discriminating genes.

The possibility of false positives might be expected when using a nominal p-value threshold of 0.05 for the initial selection of candidate discriminating genes. The subsequent steps to reduce these candidate genes to discriminating genes uses a t-statistic to test whether the sensitive/insensitive and the over/under expressed classes are statistically different. These iterative t-tests have p-values well below the nominal p-value, typically smaller than 10^-6^. Perturbation simulations based on random shuffling of discriminating gene expression values confirms the likelihood of a false positive event to be four to five orders of magnitude less than the initial nominal p-value of 0.05. This result indicates that significance values separating the four expression/sensitivity classifications are well below the nominal threshold used for their initial selection and not likely to suffer from false positives.

### Linear Discriminant Analysis (LDA) for Predicting Chemoactivity from Gene Expression

LDA can be used to assess how well discriminating genes achieve the desired goal of separating chemo-sensitive from chemo-insensitive tumor cells. A Fisher’s Linear Discriminant Analysis (DiscriminantClassification.fit Matlab 2011b) was used to determine chemoactivity prediction accuracy based on discriminating gene expressions. Classification accuracy is obtained by cross-validation using the k-fold procedure, which is based on models trained on in-fold observations to predict response for out-of-fold observations. Here cross validation was conducted using 5 folds. In this case, every training fold uses roughly 4/5 of data and every test fold uses roughly 1/5 of data. Model results are successively obtained based on excluding the first, second, third, fourth and fifth 1/5 of the dataset. In short, prediction accuracy for each observation is computed by using the model trained without this portion of the data. The reported accuracies represent the consensus score for the 5-fold cross validation models. LDA prediction accuracies will be compared between discriminating genes derived from pathway H-scores versus derived from raw correlations. The effects of data trimming on prediction accuracies will also be assessed using LDA.

### GSEA

An average of 32 (+/−9) pathways (n = 1998 nodes) are found in the topmost 10^th^ percentile of H-scores for each SOM node, with an average of 94(+/−43) pathway genes significantly correlated with each SOM NCI60 profile. GSEA is used to identify pathways that share two or more discriminating genes, to provide statistical measures for the likelihood of randomly finding shared pathway genes, and to take advantage of hooks to other databases available within the MSigDB suite of tools. A detailed description of the geneset enrichment analysis (GSEA) [27], [28] and the MSigDB can be found at http://www.broadinstitute.org/gsea/. The analysis performed here is restricted to only the KEGG, Biocarta and GO genesets. These genesets will be referred to collectively as ‘pathways’; with the caveat that the GO genesets represent an ontology, rather than biochemical pathways, as represented by the KEGG and BIOCARTA genesets. Test case results will include the standard GSEA report, consisting of i) the MSigDB pathway’s name, ii) a short description of the pathway, iii) the number of overlapping discriminating genes in the pathway and iv) the p value for statistical significance for the occurrence of this overlap across all genes in the pathway. The results presented here will include only the most statistically significant GSEA pathways (i.e. pathways having the smallest p-values). The assumption is that for each test compound, GSEA pathways with two or more discriminating genes add importance to a pathway’s role in a compound’s cell-based screening response. Furthermore, shared pathway genes may identify potential chemo-important targets. Comparisons will be provided between GSEA pathways derived from discriminating and conventionally-derived gene lists.

## Results

Test cases for 10 well-known compounds are used to illustrate this pathway-centric data mining strategy. These test cases satisfy the requirement of having structurally similar compounds (n> = 5 intra-tanimoto > = 0.7) that also appear in the same NCI60 GI50 SOM cluster. Consequently, these ten examples represent relatively well-recognized compounds, each having distinct chemoactivity profiles, which also display similar chemoactivity profiles within structurally similar groups of compounds. This design strategy is clearly chosen to avoid singletons where no structurally similar compounds share similar chemoactivity profiles. The pathway-derived discriminating genes for each test compound are analyzed through GSEA to hypothesize pathway gene-chemoactivity associations.

The results begin by summarizing the LDA accuracy for using discriminating genes to separate chemo-sensitive from chemo-insensitive tumor cells. **Table 1** lists the results of comparisons between pathway and conventionally derived discriminating genes, with and without the data trimming steps described above. These results represent the population average LDA prediction accuracies and number of discriminating genes for all 1998 GI50 SOM nodes and 2000 randomly selected NCI60 compound chemoactivity profiles. These results find that the trimmed pathway discriminating genes yield an average overall prediction accuracy of 97%, while the untrimmed, conventionally-derived discriminating genes find a mean accuracy of 82%. The results of data trimming are found to yield 3% to 4% improvements in prediction accuracy over their untrimmed counterparts, with p-values for these distribution differences of 3.7e-13 (pathway derived genes) and 3.44e-23 (conventionally derived genes). These results also indicate that pathway-derived prediction accuracies are based on fewer numbers of discriminating genes when compared to conventionally-derived discriminating genes (68 versus 300 in the trimmed cases). Data trimming eliminates an average of 24 tumor cells in the pathway and 12 tumor cells in the conventional methods.

**Table 1:** Average results for LDA analysis.

Test Case	LDA accuracy (std)	# genes(std)	# tumor cells(std)
	***Pathway***		
trimmed	0.97(0.04)	68(29)	31(8)
untrimmed	0.94(0.03)	94(43)	59(0)
	***Conventional***		
trimmed	0.86(0.04)	300(138)	41(6)
untrimmed	0.82(0.05)	382(158)	59(0)

Simulations of the pathway-centric method and the conventional correlative method were performed for all SOM GI50 nodes and 2000 randomly selected compound chemoactivity profiles. Values represent population averages and, in parentheses, their standard deviations. The average number of tumor cells in the untrimmed cases represent the full complement of NCI60 cells (Currently one cell type is no longer available for analysis), thus their standard deviation is zero.

A t-test of the distribution of LDA accuracy scores between the pathway-centric versus conventional approaches is highly significant (p<1.0e-255). A portion of this difference may be attributed directly to their discriminating genes. For example, genes significantly correlated with SOM GI50 profiles may be excluded from the pathway-derived discriminating genes, partly because their H-scores fall below the 90^th^ percentile threshold. Conversely, a portion of the pathway-derived genes do not appear in the conventionally derived genes. This result is due, in part, to the role of data trimming not finding statistical significance between the over/under-sensitive/insensitive populations for a gene set. Note that the iterative application of the Student’s t-test is based on dynamically shrinking groups of genes and tumor cells. Since the starting set of genes derived from the conventional versus pathway methods will be different, data trimming can be expected to yield differences in the final composition of discriminating genes. The most striking feature in **Table 1** is the number of genes derived from each method. Clearly the small set of pathway-derived genes yields higher LDA prediction accuracies when compared to the conventionally derived genes. Applying Occam’s razor’s principle of parsimony, the following GSEA analysis will be based on the trimmed, pathway-derived discriminating genes (i.e. minimal gene sets). The results for the 10 test cases will be summarized below. Comparisons will be provided for GSEA evaluations based on untrimmed, raw-correlation derived discriminating genes. The results for an additional eighteen test cases can be found in **Text S1 and Tables S1 & S2**.

### Case A

The results for camptothecin (CPT) find 54 discriminating gene expressions, derived from the U133A database, are needed to completely distinguish sensitive from insensitive chemoactivity. The GSEA of these discriminating genes (**Table S1**) find the KEGG and Biocarta proteasome pathways (**Table 2:** **Case A)** to be the topmost scoring pathways, while the 5^th^ ranked pathway is the Gene Ontology GO:0000502; proteasome complex. Eleven of these discriminating genes are proteasomal (**Table S1**, shown in bold) with their *over expression* corresponding to CPT *insensitivity.* Studies find that CPT activation of NF-kB [34], [35] involves degradation of its binding partner, cytosolic IkB, by the ubiquitin-proteasome pathway [36], resulting in NF-kB entry into the nucleus and promotion of CPT-induced apoptosis (i.e. CPT sensitivity). These events provide a pro-apoptotic stimulus, in support of the CPT *sensitivity* observed here to be associated with tumor cell lines exhibiting *over expression* of proteasomal genes.

**Table 2:** GSEA results for test compounds.

Geneset name	Description	# overlap genes	P value
**Case A: Camptothecin**			
Kegg proteasome	Proteasome	10	5.64E-14
Biocarta proteasome pathway	Proteasome Complex	5	4.48E-08
Macromolecular complex	GO:0032991	17	4.26E-06
Biocarta keratinocyte pathway	Keratinocyte differentiation	5	4.72E-06
Proteasome complex	GO:0000502	4	6.59E-06
**Case B: 5-fluorouracil**			
Biocarta glycolysis pathway	Glycolysis pathway	3	4.43E-06
Small nuclear ribonucleoprotein complex	GO:0030532.	3	4.11E-05
Kegg glycolysis gluconeogenesis	Glycolysis/Gluconeogenesis	4	5.71E-05
Tricarboxylic acid intermediate metabolic process	GO:0006100.	2	6.23E-04
**Case C: Colchicine**			
Oxidoreductase activity	GO:0016491	12	4.71E-07
Electron carrier activity	GO:0009055.	5	1.82E-04
Kegg regulation of actin cytoskeleton	Regulation of actin cytoskeleton	7	7.50E-04
**Case C: Combretastatin**			
Biocarta chemical pathway	Apoptotic signaling in response to DNA damage	3	1.29E-04
Biocarta BAD pathway	Regulation of BAD phosphorylation	3	2.15E-04
Kegg apoptosis	Apoptosis	4	6.68E-04
**Case D: Taxol**			
Kegg neurotrophin signalling pathway	Neurotrophin signaling pathway	7	3.53E-07
Biocarta HCMV MAPKinase pathway	Human cytomegalovirus and map kinase pathways	3	3.19E-05
Biocarta Ras pathway	Ras signaling pathway	3	8.19E-05
**Case E: Pimozide/Terfenadine/Verapamil**			
Kegg citrate cycle TCA cycle	Citrate cycle (TCA cycle)	5	4.03E-07
Kegg epithelial cell signaling in helicobacter pylori infection	Epithelial cell signaling in Helicobacter pylori infection	6	8.41E-07
Biocarta IGF1R pathway	Multiple antiapoptotic pathways involving BAD phosphorylation	4	4.10E-06
Biocarta Ras pathway	Ras signaling pathway	4	4.10E-06
**Case F: Purvalanol**			
Pore complex	GO:0046930	7	7.05E-10
Nuclear envelope	GO:0005635.	8	5.38E-09
Nuclear pore	GO:0005643.	6	1.22E-08
Nuclear membrane part	GO:0044453	6	9.41E-08
Nuclear membrane	GO:0031965.	6	2.90E-07
Envelope	GO:0031975	9	3.75E-07
**Case G: Dasatinib**			
Cell cycle GO:0007049	GO:0007049.	17	2.64E-11
Cytoskeletal part	GO:0044430.	13	6.68E-09
Kegg cell cycle	Cell cycle	10	1.96E-08
Kinesin complex	GO:0005871.	5	2.98E-08
Microtubule associated complex	GO:0005875.	7	3.36E-08
Regulation of cell cycle	GO:0051726.	11	3.96E-08
Microtubule motor activity	GO:0003777.	5	6.42E-08
Mitotic cell cycle	GO:0000278.	10	7.88E-08
Cytoskeleton	GO:0005856.	14	1.82E-07
Cell cycle process	GO:0022402.	10	7.37E-07

The GSEA results for each test compound include; column one: geneset name as it appears in MSigDB, column two: pathway description, column three: number of discriminating genes that are shared in each respective pathway, column four: statistical significance for the occurrence of these overlapping genes for each geneset. Test cases appear in the order presented in the Results. These results are based on analysis of the U133A dataset.

The discriminating genes associated with CPT *insensitivity* (rows 1–16 of **Table S1**) include the nuclease MRE11 (ranked 4^th^ in Table S1), with its overexpression associated with CPT insensitivity. Topoisomerases generate transient covalent phosphotyrosine intermediates with DNA [37]. CPT traps these intermediates to produce DNA damage via strand breaks. Nucleases repair topoisomerase-mediated DNA damage by removing topoisomerases covalently bound to DNA [38]. MRE11 over expression, as a means to enhance repair of CPT-induced DNA damage, could be hypothesized for the CPT insensitive subset of tumor cell lines. Additionally, CPT’s over expressed discriminating gene include pro-survival genes EIF4G1 and BCL2L1, and mitogen-activated protein kinases (including MAPK13 and MAPK14, ranked 2^nd^ and 14^th^ in **Table S1**), each involved in cellular survival and proliferation-inducing processes. Taken together, their *over expression* is consistent with the observed CPT GI50 *insensitivity* within selected tumor cells. Collectively, CPT’s pathway gene-chemoactivity associations provide a literature-validated rationale for cellular CPT sensitivity as well as hypothesizing a genetic basis for CPT insensitivity.

Analysis of the U133Plus dataset finds that overexpressed discriminating genes associated with CPT sensitivity identify the GSEA pathways Biocarta HER2, PDG, EGF and MAPK signaling pathways, and the GO protein kinase inhibitor (GO:0004860), kinase inhibitor (GO:0019210) and protein kinase regulator activity (GO:0019887) pathways. These results are consistent with the finding that MAPK signaling is associated with apoptotic cell death by camptothecin [39]. The U133Plus-derived GSEA pathways corresponding to CPT insensitivity are associated with over expression of ATPase synthase (GO:0016469; proton transporting two sector ATPase complex, GO:0031966; mitochondrial membrane, GO:0005740; mitochondrial envelope, GO:00031090; organelle membrane, GO:0005743; mitochondrial inner membrane), with ample evidence of the ATPase synthase being associated with drug resistance [40]. Here the proteasomal pathways implicated from the U133A gene expressions are not evident from the U133Plus gene expressions. A close inspection of the origins of this difference appears to reflect true differences in each platform’s gene expression measurements. Comparisons of proteasomal gene expressions between the U133Plus versus U133A platforms find them to be very poorly correlated (r_ave_ = 0.52;std = 0.21); a result that most likely contributes to the identification of alternative chemo-important pathways when using the U133Plus dataset.

The GSEA results for CPT can be used to address whether corresponding results are obtained from Ingenuity Pathway Analysis(IPA). Using CPT’s discriminating genes, the top scoring IPA canonical pathway is the Protein Ubiquitination Pathway, comprised exclusively of proteasomal members. While the top scoring IPA networks are Cell Death, Genetic Disorder and Neurological Disease: also with a strong representation of proteasomal members. The two top scoring IPA functions are Developmental Disorder and Dermatological Diseases and Conditions, with a sparse representation of proteasomal members. Thus GSEA and IPA results find strong agreement with their best scoring entries. Inspection of lower scoring results for both methods identify many additional roles associated with CPT’s discriminating genes. While the IPA results may lead to alternative classes of cellular mechanisms associated with discriminating gene lists, the current analysis will focus only on the top scoring entries generated by GSEA.

CPT’s GSEA pathway results can also be compared to application of conventional (i.e. nonpathway-centric) methods to generate chemo-important genes. Using the NCI60 GI50 profile for CPT, Pearson correlation scores and their significance values (p-values) can be determined for all NCI60 gene expressions. The top-most significant correlations yield 34 genes for CPT. Consistent with the results of Nakatsu et al. [18], CPT’s known target, topoisomerase 1, is absent from this gene set. The GSEA results identify Chromosome (GO:0005694), Kegg Parkinsons Disease, Kegg Notch Signalling, Kegg WNT Signaling and Kegg Alzheimers disease as the five best scoring GSEA pathways. Noteworthy is that increasing the correlation threshold sufficient to yield a set of 120 discriminating genes produces GSEA pathways that include Kegg Proteasome, Biocarta Proteasome and Proteasome Complex among its most significant pathways; albeit at a cost of nearly four times more discriminating genes when compared to the pathway-derived method.

### Case B

These results for 5-flurouracil (5-FU) find its SOM GI50 profile appearing in a neighboring, yet distinct, SOM cluster from that of CPT. 5-FU’s discriminating genes find pathways involved in glycolysis as the 1^st^, 3^rd^ and 4^th^ most significant GSEA hits (**Table 2:** **Case B**). The role of glycolysis in 5-FU efficacy finds the sensitivity of gastric cancer to 5-FU treatment to be related to the rate of glucose transport [41]. Two of 5-FU’s discriminating genes, glyceraldehyde-3-phosphate dehydrogenase (GAPDH) and phosphoglycerate kinase 1 (PKG1), appear as the most over expressed genes for cells sensitive to 5-FU, while over expression of discriminating genes for mitogen activated protein kinases (MAPK13 and MAPK14) and eukaryotic translation initiation factor genes (EIF1AP1 and EIF4A2) correspond to 5-FU insensitivity; leading to the hypothesis that efficacy of 5-FU may depend on over expression of glycolytic genes and under expression of proliferation-related genes.

In distinct contrast to the U133A-derived results, the U133Plus gene expressions find nine of 5-FU’s topmost scoring GSEA pathways to be associated with the plasma membrane. In support of this finding, a nearly 2-order of magnitude difference is found between IC_50_ values of 5-FU versus FuDR and FUR [42]. This difference has been associated with the greater capacity to transport 5-FU across the plasma membrane of tumor cells, and has led to the proposal that inhibitors of membrane transport may enhance 5-FU efficacy [43], [44]. Thus the GSEA glycolytic pathways identified with the U133A gene expression data are no longer in the topmost scoring pathways determined from the U133Plus dataset. Glycolytic pathways are necessarily dependent on plasma membrane transport of glucose, thus the role of the plasma membrane is implicated from both gene expression datasets as a component of 5-FU chemoactivity. Although the origins of these differences may reflect, at a minimum, biological variation, they are also an indication that methodologies such as proposed here can benefit from the analysis of greater numbers of high quality gene expression measurements.

Using conventional gene expression-GI50 Pearson correlations finds 242 genes in the top 10^th^ percentile. The top ten GSEA pathways for these genes find no overlap with those listed for **Table 2:** **Case B**. However, the 6^th^ ranked GSEA pathway is GO:0006007: Glucose Catabolic Processes, based on the presence of phosphogluconate dehydrogenase (PGD) in its correlated gene list. Thus conventional analysis also suggests a role for glucose metabolic pathways in 5-FU chemoactivity.

### Case C

Case C consists of compounds known to target tubulin. One example from this group is colchicine, where its top scoring GSEA pathways are related to oxidation-reduction (**Table 2:** **Case C Colchicine**). Literature validation of this result finds the functions of actin and tubulin to be redox-regulated [45], [46]. The sulfhydryl groups in tubulin affect assembly and are under control of thioredoxin or glutaredoxin systems. siRNA knockouts of glutaredoxin exhibit hampered actin assembly [47]. The results reported here find two of cholchicine’s discriminating genes, thioredoxin interacting protein (TXNIP) and thioredoxin reductase 1(TXNR1), to be most positively correlated with the SOM GI50 profile containing colchicine. Additional positively correlated discriminating genes include malate dehydrogenase (MDH1), isocitrate dehydrogenase (IDH1) and cytochrome c oxidase subunit VIIb (COX7B), all genes within the Oxidoreductase Activity pathway (GO:0016491). The 4^th^ ranked GSEA pathway is KEGG regulation of actin cytoskeleton, also consistent with the role of oxidation-reduction on actin assembly. These U133plus gene expressions support this finding by identifying oxidoreductase pathways within its topmost GSEA results. Here, as with the cases to follow for taxol and combrestatin, the baseline expression of colchicine’s putative target, tubulin, appears to play a less important role in chemoactivity when compared to redox-related cellular processes.

Interestingly, combretastatin occupies a colchicine-neighboring SOM cluster, yet yields a different set of GSEA pathways. Its top scoring GSEA pathway (**Table 2:** **Case C Combretastatin**) is the Biocarta Chemical Pathway: apoptotic signaling in response to DNA damage. Literature validation of this result finds that proliferating human endothelial cells exhibit internucleosomal DNA fragmentation when incubated with combretastatin A-4 [48]. Combretastatin’s 2^nd^ ranked GSEA pathway involves regulation of BAD phosphorylation. Included in the 15 over regulated discriminating gene expressions associated with combretastatin sensitivity are BAD, IkBkB (inhibitor of kappa light polypeptide gene enhancer in B-cells, kinase beta) and CDC2L1(cell division control like 1), all of which are involved in signaling events associated with BAD phosphorylation.

While the U133Plus results for combretastatin did not identify the Biocarta Chemical Pathway as found with the U133A dataset, ‘chemical biosynthetic processes’ are indicated within 8 of its 10 top most scoring GSEA pathways, most of which relate to biosynthetic processes involving the formation of cytokines. Recent studies find that the induction of vessel narrowing, hypoxia, and hemorrhagic necrosis in murine mammary tumors by vascular disrupting agents, such as combretastatin A4, is accompanied by elevated tumor levels of the chemokine CXCL12 and tumor cell repopulation [49]. In this instance, the additional gene expression data provided in the U133Plus platform yielded chemical reaction pathways involved in combretastatin chemoactivity that place a higher priority on processes involved in chemokine production rather than chemical pathways related to DNA damage. Both results find support within the literature. Consistent with the results to follow for taxol, although combretastatin also targets tubulin, its cellular sensitivity appears to be consistent with the expression of non-tubulin related genes.

The conventional analysis finds 240 genes in the upper 10^th^ percentile of correlations for colchicine and combretastatin A4. GSEA of these colchicine genes yields KEGG Bladder Cancer, Membrane Organization and Biogenesis (GO:0016044), Biocarta Defragmentation Pathway, Cell_Cortex (GO:0005938) and Mitochondrial Membrane Organization and Biogenesis (GO:0007006) as its topmost pathways. Here evidence for membrane organization is indicated, rather than redox related processes listed in **Table 2:** **Case C**. GSEA analysis of the combretstatin A4 correlated genes finds the top five pathways to include KEGG Thyroid Cancer, Nuclear Pore (GO:0005643), Pore Complex (GO:0046930), KEGG Adherens Pathway and Induction of Apoptosis by Intracellular Signalling (0008629). These results point to distinctly different cellular mechanisms from those listed in **Table 2:** **Case C**. Noteworthy for designing experiments in support of these conventional-based pathways is the existence of an order of magnitude more genes to be studied when compared to the pathway-centric approach.

### Case D

The next test compound is taxol. The neurotrophin signaling pathway (**Table 2:** **Case D**) ranks at the top of the GSEA pathways for taxol, with 7 (AKT1, MAP2K1, IRS1, IRAK1, RPS6KA3, CSK and PSEN1) of its 34 discriminating genes appearing in this pathway. Neurotoxicity is a well-known complication of taxol therapy [50] and up-regulation of all of these seven genes is associated with taxol sensitivity; with the KEGG Neurotrophin signaling pathway at the top of the list. In addition, taxol-induced apoptosis depends on MAP kinase pathways [51]. The 2^nd^ ranked pathway, Human Cytomegalovirus and Map Kinase Pathways, is particularly interesting in light of the observed reversal of taxol resistance due to involvement of the PI-3 kinase-AKT 1 pathway [52]. Whereas the 3^rd^ ranked pathway, Biocarta Ras pathway, finds sensitivity associated with over expression of AKT1 (v-akt murine thymoma viral oncogene homolog 1), MAP2K1 (mitogen-activated protein kinase kinase 1) and CASP9 (caspase 9, apoptosis-related cysteine peptidase), also involved in the PI-3 kinase-AKT 1 pathway. The pathway gene-chemoactivity results point directly to the importance of taxol-related neurotoxicity, also validated in literature reports.

The pathway results for taxol using the U133Plus gene expressions appears, initially, to not support the U133A-derived results. The GSEA results for the U133Plus gene expressions identify six GSEA pathways associated with the cell cycle: Biocarta G2 Pathway; cell cycle, GO:0000082; G1-S transition of mitotic cell cycle, GO:0051329; interphase of mitotic cell cycle, GO:0000278; mitotic cell cycle, GO: 0022403, cell cycle phase, GO:0051325, interphase) and five GSEA pathways associated with amino acid metabolism (GO:0015171; amino acid transmembrane transporter activity, GO:00022804; active transmembrane transporter activity, GO:0005275; amine transmembrane transporter activity, GO:0046943; carboxylic acid transmembrane transporter, GO:0005342; organic acid transmembrane transporter activity). Taxol’s role in mitotic blockage was reported very shortly after its discovery [53]. In addition, the activities of selected amino acid transporters have been recently proposed as biomarkers for assessing the response to taxol treatment [54]. While the U133A dataset identified neurotrophin signaling as an important, and well-published, component of taxol chemoactivity, the U133Plus dataset finds additional roles for multiple pathways involved in the mitotic cell cycle and amino acid metabolism. Both results are an important component of taxol’s chemoactivity-pathway gene associations. As with the two previously discussed agents that target tubulin, non-tubulin related gene expressions and their associated pathways appear to contribute to the overall cellular response. The genes generated using conventional chemoactivity-gene expression correlations do not find Neurotrophin Signalling or Cell Cycle in their top 50 GSEA pathways.

**Figure 4:**
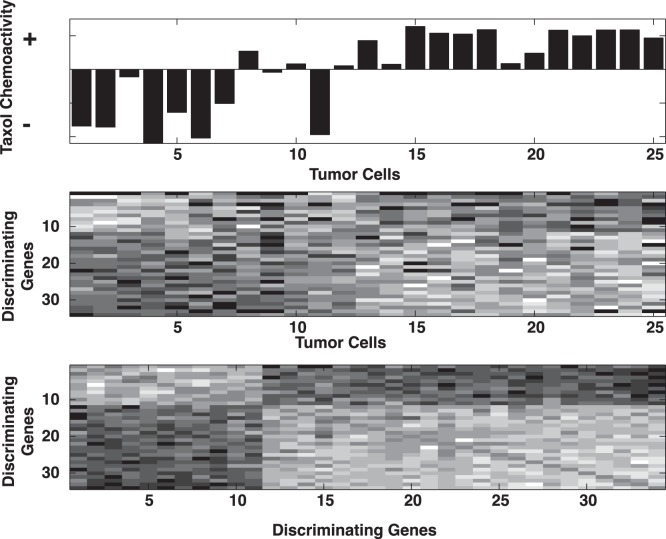
Illustrations of Taxol’s discriminating genes. Top panel displays Taxol’s chemoactivity profile (+:sensitive −::insensitive) for its 25 discriminating genes, derived from the U133A dataset. The middle panel displays the relative gene expressions (light:over dark:under) for these same tumor cells. The lower panel displays the Pearson correlation scores (positive:light negative:dark) for taxol’s discriminating genes.

Taxol’s discriminating genes can be examined for associations between gene over and under expression and chemoactivity. The top panel in **Figure 4** displays Taxol’s reference chemoactivity for the subset of 25 tumor cells used to establish its discriminating gene set. The sensitive(+) and insensitive(−) cellular responses are grouped at the right and left, respectively. The middle panel displays the relative expressions (light:over dark:under) for taxol’s discriminating genes across these same 25 tumor cells. The correspondence between expression and chemoactivity is evident with over expressed genes (rows 1–11) and taxol insensitive tumor cells (columns 1–11) appearing in the upper left quadrant, while over expressed genes (rows 12–34) and taxol chemo-sensitive tumor cells (columns 12–25) appearing in the lower right quadrant. The lower panel emphasizes these differences in gene expressions by displaying the Pearson correlation values (positive:light negative:dark) for these 34 discriminating gene expressions. Clearly, expressions for discriminating genes 1–11and 12–34 represent distinguishable patterns. These gene expressions differences may serve as potential biomarkers of taxol chemoactivity.

### Case E

The antiproliferative actions of pimozide, terfenadine and verapamil are attributed to the initiation of apoptotic cell death pathways [55] resulting from their effects on Ca^2+^channels. The GSEA analysis (**Table 2:** **Case E**) for these agents identifies the TCA-cycle as an important pathway. Mitochondrial calcium ions promote a number of events that sustain ATP levels in the cell. The well-known Warburg effect identifies a switch from aerobic and anaerobic sources of ATP production [56]. Specifically, in the absence of calcium transfer cells use autophagy to sustain survival, while cells performing under aerobic metabolism utilize the TCA cycle as their primary source of ATP. Cells exhibiting over expression of TCA-related metabolism genes would be quite sensitive to calcium perturbations. Consistent with this premise, tumor cells most sensitive to these agents exhibit over expression of TCA-related discriminating genes, IDH3A, IDH1, IDH3G, ACO2 and ACLY. The observation of cells insensitive to these agents and having under expression of these TCA-related genes may reflect the role of autophagy in their survival.

The U133A and U133Plus gene expressions for pimozide offer complementary hypotheses about the role of calcium flux on glucose metabolism. The U133Plus results find MAPK signaling as its topmost scoring GSEA pathways. Studies have shown that glucose metabolism is controlled by a combination of allosteric activators and inhibitors that provide a variety of potential targets for MAPK regulation [57]. For example, glucose uptake and glycolysis is dependent on ERK signaling within MAPK pathways, providing a point of coordinated control on glucose metabolism [58]. Taken together, the U133A and U133Plus results hypothesize a role for MAPK signaling in aerobic glycolysis, and implicate agents that affect calcium flux as influencing this process. The top scoring GSEA pathways derived from conventional gene lists are Biocarta Rho pathway, Biocarta integrin pathway, electron carrier activity (GO:0009055), Biocarta gleevec activity and Biocarta ECM pathway. Again, sharing no overlay with the pathways listed in **Table 2:** **Case E**. However, the role of calcium in these pathways would be an indication for further study.

### Case F

Cyclin-dependent kinases (CDKs) control cell cycle progression [59], [60] and have become attractive targets in the search for small molecular weight inhibitors of the cell cycle. p42/p44 MAPKs are intracellular targets of the CDK inhibitor purvalanol [61]. The GSEA results for purvalanol (**Table 2:** **Case F**) point to the nuclear pore and pore complex as important pathways. The number of nuclear pore complexes (NPCs) nearly doubles during interphase in dividing cells. Although the coordination of this event within the cell cycle is poorly understood, it appears that CDKs, especially Cdk1 and Cdk2, promote interphase NPC formation in human dividing cells [62]. Consistent with this literature report, CDK inhibition disturbed proper expression and localization of some nucleoporins, which trigger post-mitotic NPC assembly. The discriminating genes for purvalanol include RANBP2 (RAN binding protein 2), IPO7 (importin 7), NUTF2 (nuclear transport factor 2), KPNA3 (karyopherin alpha 3 (importin alpha 4)), NUP153(nucleoporin 153kDa), NUP88 and BAX (BCL2-associated X protein), with their under expression correlating with purvalanol sensitivity. Here the role of CDK inhibition apparently manifests itself by inhibiting the formation of NPCs necessary to satisfy the requirements for successful passage through interphase. The U133Plus-derived results also implicate MAPK pathway members as targets, by finding the KEGG MAPK Signaling pathway as purvalanol’s topmost scoring GSEA pathway, at a remarkably low p-value of 5.83×10^−12^. This result is due mostly to having 26 MAPK pathway genes included within purvalanol’s discriminating genes. Clearly, both gene expressions find pathways implicating a role of purvalanol in CDK inhibition. Genes selected by conventional analysis find no GSEA pathways related to nuclear pore formation or MAPK signaling.

### Case G

Dasatinib, a potent inhibitor of SRC (SaRComa) family kinases, is currently under development against a variety of tumor types [63]. SRC is a member of the tyrosine kinase family of proteins that function to transfer a phosphate group from ATP to the tyrosine residue of a protein. Phosphorylation of proteins by kinases is important to cellular communication and regulation of cellular activities such as cell division. The GSEA results for dasatinib (**Table 2:** **Case G**) find cell cycle in five of the ten most statistically significant GSEA pathways, with cytoskeletal/kinesin/microtubule related pathways comprising the remainder of the list. These results are consistent with the role of SRC inhibition by dasatinib on the cell cycle. The existence of pathways involving motor elements is also consistent with cellular events, such as movement along a microtubule, that are coupled to the hydrolysis of ATP. Included in the discriminating genes that are positively associated with dasatinib sensitivity are PTK2, RAF1 and PLK4. PTK2, is a member of the focal adhesion kinase (FAK) subfamily of protein tyrosine kinases, and is a known target of dasatinib [64]. RAF1 and PLK4 are members of the serine/threonine family of protein kinases which also function in the cell cycle. Dasatinib treatment has been found to inhibit other serine/threonine kinases including AKT and ERK1/ERK2 [65]. Interestingly over-expression of the tyrosine kinase c-SRC (also known as CSK) is associated with dasatinib insensitivity. c-SRC is capable of phosphosphorylating a negative regulatory site on tyrosine kinase family members. The observation that dasatinib is also known to target c-SRC raises the possibility that inhibition of a key negative regulatory element of SRC proteins may be responsible for the lack of dasatinib sensitivity to tumor cells over expressing c-SRC. In support of this possibility is the finding that mutated c-SRC is not capable of SRC suppression [66].

Analysis using the U133Plus gene expressions expands this result by including JUN (JUN oncogene), MAPK8 (mitogen activated protein kinase 8), SRC (v-SRC sarcoma) and FYN (FYN oncogene related to SRC) as protein kinases within dasatinib’s discriminating genes. The topmost GSEA pathways now include the Biocarta Integrin pathway, Biocarta AT1R pathway, KEGG adherens pathway and Biocarta Cell2Cell pathway, all containing c-SRC and the known target of dasatinib, v-SRC. In addition, the U133A-derived role of the cytoskeleton in dasatinib’s activity, via coupling to ATP hydrolysis, is further reinforced by finding U133Plus-derived GSEA pathways that modulate ATP hydrolysis by proteins from pathways involved in oxidoreductase activity. Conventional analysis of U133A gene expressions correlated with dasatinib chemoactivity find KEGG Huningtons Disease, KEGG Parkinsons Disease, KEGG Oxidative Phosphorylation, KEGG Alzheimers Disease and Organelle Inner Membrane (GO:0019866) as the top scoring GSEA pathways. Here the lack of cell-cycle pathways would not be consistent with the literature supported pathways described from the pathway-centric analysis.

**Figure 5:**
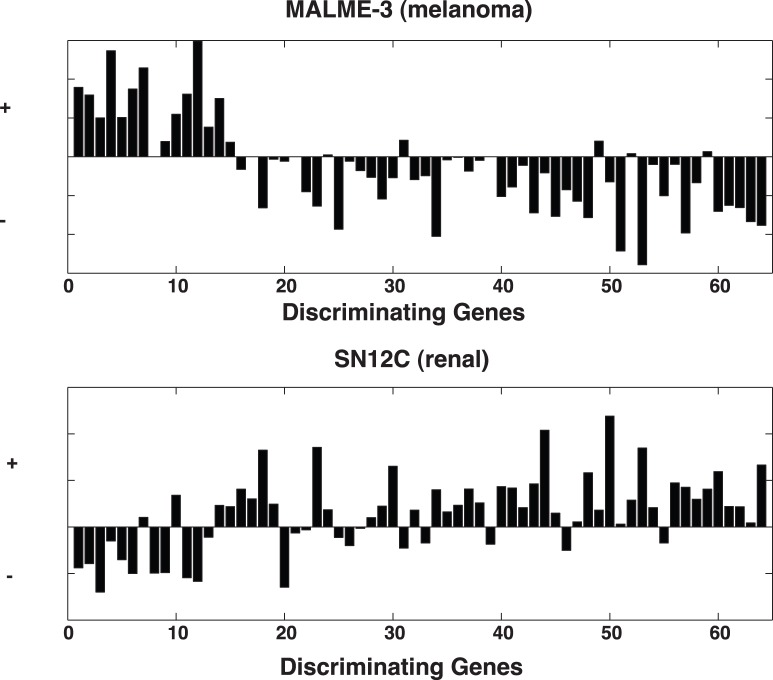
Discriminating gene expressions for MALME-3 (top panel) and SN12C (bottom panel). Over and under expressed genes correspond to upward and downward directed bars, respectively. Genes are divided from left to right into groups of 15 and 50 genes, respectively. An average over expression of genes in the first group and under expression of genes in the second group corresponds to MALME-3 chemo-insensitivity. The convers holds for SN12C, where an average under expression of genes in the first group and over expression of genes in the second group correspond to chemo-sensitivity.

The pathway-centric speculations regarding the gene expressions involved in the chemoactivity of dasatinib can serve as a basis for proposing further experimental testing. **Figure 5** displays bar graphs for discriminating gene expressions of a dasatinib insensitive (MALME-3) and sensitive (SN12C) tumor cells. Here dasatinib’s 65 discriminating gene expressions fall into two populations; a group of 15 genes, at the left, and a group of 50 genes, at the right. Over expression in the smaller group and under expression in the larger group appears to be consistent with dasatinib insensitivity in the MALME-3 cell line, while expressions in the opposite directions for these groups appears to be consistent with sensitivity for the SN12C cell line. These indirectly-derived results can be independently tested in other tumor cell lines by examining their differential in gene expressions between these sets of 15 and 45 genes. The task of finding other tumor cells that are concordantly similarly expressed within each group and concordantly oppositely expressed amongst others may prove difficult. Alternatively, gene knock-outs/−ins may be employed on the existing cells to modulate the extent of differential in gene expression. Results from each of these strategies may also prove difficult to interpret due to the complex roles of multiple genes in higher order cellular networks. A third alternative strategy would attempt to determine a direct interaction between dasatinib and one of these discriminating genes (or gene products) using x-ray crystallographic and NMR methods [67], affinity chromatography [68], [69] or protein microarrays [70].

### Supplementary Test Compounds

The Supplementary Test Compounds section in Text S1 and Table S2, includes results for an additional 18 test compounds, the GSEA pathways identified by their discriminating gene and supporting literature validations. In principal, each SOM node can yield a set of discriminating genes that can be associated with its cluster members. Selecting an appropriate SOM node will depend, in part, on special interest in a set of compounds or a set of discriminating genes.

## Discussion

The method presented here represents a pathway-centric perspective for assigning the functional relevance of constitutive gene expressions from untreated tumor cells to mechanisms important for *in vitro* chemical inhibition of tumor cell growth. Publicly available databases obtained from the NCI60 and a pathway-centric means to identify discriminating genes as input to GSEA were used to formulate hypotheses about a compound’s pathway gene-chemoactivity associations. The results, reported for a set of well-known compounds screened in the NCI60, typically yield a small set of statistically significant GSEA pathways (usually on the order of 5 pathways) associated with each compound’s literature-supported pathway targets. In a few cases, actinomycin D:TOPO2A (supplementary test case, Table S2) and dasatinib:PTK2, extremes in gene expression for a compound’s putative molecular target were found. The more common finding reveals an important role for over and under expression of small groups of discriminating, off-target genes in a compound’s cellular response, inclusive of intended effects as well as side effects. These results emphasize the importance of comprehensive examinations into constitutive gene expressions and their associated cellular pathways for roles in *in vitro* chemoactivity. The methods are general and can be applied to analyze preclinical databases similar in design to the NCI60.

Further support for applying data mining efforts that use multiple chemoactivity versus individual chemoactivity-gene expression correlations can be found in Lee et al.’s [71] examination of correlations between NCI60 chemoactivity and gene expression [72], [73]. Their analysis finds that single-event correlations, as in a distinctly high correlation value for only one chemoactivity-gene expression profile, may be due to experimental noise. Alternatively, families, typically of ∼25 compounds, are correlatively associated with a single gene’s expression profile. Following their strategy, but reversing the question, finds (unpublished by author) that around 15 gene expressions are significantly correlated with a chemoactivity profile. This finding favors a strategy that examines groups of gene expressions, as is done here with the pathway-centric approach, rather than the more conventional single-event method. Nonetheless, the conventional and pathway-centric approaches should be considered for these types of analysis. Clearly, resolving whether one approach might be favored over another will require extensive experimental testing.

The pathway-centric method proposed here bears conceptual similarities to the Connectivity Map (CM) method proposed Lamb *et al.* [11], [12], and with the work by Sirota *et al.* [10]; representing, respectively, procedures that use gene expression-chemoactivity associations to relate existing preclinical small molecules to their cellular mechanism of action, or to their potential therapeutic use against alternative diseases. The CM analysis uses measures of over 7000 gene expressions and chemoactivity responses for tumor cells exposed to 1309 agents (CM build 02); with a strong overlap in cell types and compounds with the NCI60. ‘Signature’ genes proposed for CM analysis represent instances of large expression shifts following drug exposure to tumor cells. The CM chemoactivity and gene expression databases are scanned for the occurrence of compounds and genes that also possess extreme values for the test set of ‘signature’ genes. The procedure of Sirota *et al.* [10] also utilizes CM’s compound database, however, ‘signature’ genes are scanned across Gene Expression Omnibus [32], [74] gene expressions associated with 100 diseases (cancer and non-cancer). In contrast, the method proposed here is based on baseline gene expressions measured from 60 untreated cancer-only diseases and a compound database of over 60k agents. While the methods of Lamb *et al.* [11], [12] and Sirota *et al.* [10] and the pathway-centric approach proposed here share the goal of identifying associations between gene expressions and chemosensitivity, they differ primarily in the derivation of ‘signature’ genes versus ‘discriminating’ genes, in the use of gene expressions from untreated tumor cells, and in the application of pathway information into the data analysis.

### Summary Comments

The use cultured tumor cells for explorations into the genetic basis of drug activity represent one preclinical strategy for exploring various aspects of cancer biology, despite their limited ability to reflect responses in the human body [75]. Computational strategies, such as presented here, must be viewed with a high degree of skepticism until further validations become available using alternative preclinical models. Unfortunately, the existence of potentially many off-target effectors as important to chemoactivity will present considerable experimental challenges for hypothesis testing. In lieu of these studies, additional confidence in these data mining results may be gained by examining consistency. Once again, the CPT example is used for illustration. Compounds with GI50 profiles *inversely* correlated with CPT chemoactivity can be examined to assess whether their results also point to pathways consistent with those found for CPT. The most negatively correlated SOM GI50 profile to that containing CPT is the SOM GI50 profile containing parthenolide (PN). The GSEA pathways for both CPT and PN (see **Supplemental Test Compounds, Text S1 and Table S2,** for the PN GSEA results) find important roles for the proteasome (PSM). However, examination of PN’s discriminating genes finds that over expression of PSM genes corresponds to CPT sensitivity and PN insensitivity. Gupta *et al.* [76] found that p53 can protect cells against CPT-induced cytotoxicity, leading to their rationale for the selectivity of CPT towards tumors with p53 mutations. PN has been found to promote ubiquitination of MDM2 and *activation* of p53 cellular functions [77]. MDM2 is ubiquitin ligase that promotes proteasomal degradation of numerous proteins, including itself. p53, a substrate of MDM2, functions as part of a regulatory feedback loop involved in cancer-related pathways, notably tumor suppression. Levels of p53 are tightly regulated by MDM2, whereby elevated levels of p53 activate MDM2 expression, which in turn sequesters p53, ubiquinates it and marks it for proteasomal degradation. Thus PN’s role in activating p53 cellular functions can be hypothesized to reduce CPT sensitivity, an effect consistent with the inverse correlations between CPT and PN chemoactivity profiles. This line of reasoning can be taken one step further. Recall that the role of PSM genes in CPT’s sensitivity was found, via GSEA analysis, to involve NF-kB. Recently it has been shown that activation of NF-kB by chemotherapeutic agents was found to protect cells from apoptosis. Sharma *et al.* [78] tested the hypothesis that inhibition of NF-kB-mediated gene transcription may sensitize tumor cells to chemotherapeutic agents. They find that an imidazoline NF-kB inhibitor sensitizes leukemic T cells to CPT. Elucidation of the potential cellular mechanism revealed that imidazoline prevents nuclear translocation of NF-kB. These findings are consistent with this report’s hypothesis of NF-kB mediating CPT sensitivity. Collectively these results lend confidence that the approach proposed here is yielding a consistent picture, at least for CPT, of important causal relationships between chemoactivity and constitutive gene expressions.

While the proposed methodology has yielded qualitatively similar results when using gene expressions derived from two measurement platforms, assessments of how robust these pathway-centric results may be, regardless of the methods used to quantify gene expressions, cannot be adequately assessed here. For the most part, chemo-important pathway results are in general agreement when derived from the U133A and U133Plus datasets. Cases that differ find their origins to be due, in part, to differences in gene expressions. A global inspection of the pairwise Pearson correlations between gene expressions derived from the U133A versus U133Plus platforms finds an average correlation value of 0.68 (std = 0.19). Thus only slightly less than half (r^2^ = 0.46) of the measurement variation between platforms can be explained by a simple correlative model. The above comparisons of results using U133A and U133Plus measurements finds that, at least for these test compounds, the agreement between pathway gene expressions and chemoactivity appears to be sufficient to yield qualitatively similar GSEA pathways. Instances, such as the failure of the U133Plus gene expressions to identify the U133A-derived proteasomeal pathways, can be assessed with respect to the effects of noise contamination on measurement signal. Using the CPT example and the U133A gene expressions, simulations were conducted to determine how well the proposed methodology tolerates poorly concordant gene expressions. Application of white noise sufficient to degrade the U133A gene expression measurements to yield an average pairwise Pearson correlation on the order of r = 0.6 was sufficient to eliminate the GSEA proteasomal pathways from the topmost scoring hits for CPT. The proposed methodology, however, provides a symptomatic indicator of the effects of data noise by finding that, again with the CPT example, the number of discriminating genes is reduced by nearly half when compared to the original gene set when using white-noise perturbed expression measurements. This reduction is expected due to a lower number of significant pathway H-scores resulting from greater randomness in the expression data. While these results suggest that the proposed methodology is modestly tolerant of measurement variations across gene expression platforms, a detailed examination using numerous publically available gene expression datasets remains part of future studies. Based on the above results, increased numbers of gene expression measurements, as afforded by the U133Plus over the U133A datasets, appears to provide greater numbers of GSEA pathway hits that also share a common theme with respect to biological processes. These redundancies in GSEA pathways reinforce support for the importance of a proposed pathway’s gene expressions on chemoactivity. It is important to stress that the results reported herein for gene expression measures using either the U133A or U133Plus platforms hypothesize GSEA pathways that are each reasonably well supported in literature reports. Instances where GSEA results differ according to measurement platform suggest caution, when considering results obtained from only one set of gene expressions, and encourage consensus assessments of results using gene expression derived from multiple platforms. Experimental testing of hypothesized pathway gene-chemoactivity associations, regardless of the source of gene expression measurements, will be a necessary step for validation.

## Supporting Information

Table S1 **U133A-derived genes selected to discriminate sensitive versus insensitive tumor cell responses to CPT.** Genes are ordered from top to bottom according to correlation strength. Top 16 genes represent discriminating genes with expressions negatively correlated to CPT’s SOM NCI60 GI50 profile, where over expression corresponds to chemo-insensitivity. The bottom 38 discriminating genes have expressions positively correlated to CPT’s SOM NCI60 GI50 profile, with over expression corresponding to CPT chemo-sensitivity. Proteasomal genes are highlighted in bold.
(DOC)

Table S2 **GSEA Results for Supplemental Test Compounds.**
(DOC)

Text S1 **The Supplementary Test Compounds section in Text S1 and Table S2, includes results for an additional 18 test compounds, the GSEA pathways identified by their discriminating gene and supporting literature validations.** In principal, each SOM node can yield a set of discriminating genes that can be associated with its cluster members. Selecting an appropriate SOM node will depend, in part, on special interest in a set of compounds or a set of discriminating genes.
(DOC)
