# Supplementary material for: Integrating Constitutive Gene Expression and Chemoactivity: Mining the NCI60 Anticancer Screen
Source: PLoS One. 2012 Oct 2;7(10):e44631. doi: 10.1371/journal.pone.0044631 (PMC3462800; doi:10.1371/journal.pone.0044631)
Supplement: Table S1 — U133A-derived genes selected to discriminate sensitive versus insensitive tumor cell responses to CPT. Genes are ordered from top to bottom according to correlation strength. Top 16 genes represent discriminating genes with expressions negatively correlated to CPT’s SOM NCI60 GI50 profile, where over expression corresponds to chemo-insensitivity. The bottom 38 discriminating genes have expressions positively correlated to CPT’s SOM NCI60 GI50 profile, with over expression corresponding to CPT chemo-sensitivity. Proteasomal genes are highlighted in bold. (DOC) [file pone.0044631.s001.doc]

**Supplemental Table S1**. U133A-derived genes selected to discriminate sensitive versus insensitive tumor cell responses to CPT. Genes are ordered from top to bottom according to correlation strength. Top 16 genes represent discriminating genes with expressions negatively correlated to CPT’s SOM NCI60 GI50 profile, where over expression corresponds to chemo-insensitivity. The bottom 38 discriminating genes have expressions positively correlated to CPT’s SOM NCI60 GI50 profile, with over expression corresponding to CPT chemo-sensitivity. Proteasomal genes are highlighted in bold.

|  | **Discriminating genes with expressions negatively correlated to CPT’s SOM NCI60 GI50 profile** |
| --- | --- |
| ATP6V1A | ATPase,_H+_transporting,_lysosomal_70kDa,_V1_subunit_A |
| MAPK13 | mitogen-activated_protein_kinase_13 |
| ACO2 | aconitase_2,_mitochondrial |
| MRE11A | MRE11_meiotic_recombination_11_homolog_A_(S._cerevisiae) |
| PSEN1 | presenilin_1_(Alzheimer_disease_3) |
| OSBPL3 | oxysterol_binding_protein-like_3 |
| EIF4G1 | eukaryotic_translation_initiation_factor_4_gamma,_1 |
| BCL2L1 | BCL2-like_1 |
| NCKAP1 | NCK-associated_protein_1 |
| DUSP6 | dual_specificity_phosphatase_6 |
| SORL1 | sortilin-related_receptor,_L(DLR_class)_A_repeats-containing |
| SF1 | splicing_factor_1 |
| HIST1H2BK | histone_cluster_1,_H2bk |
| TRAF4 | TNF_receptor-associated_factor_4 |
| MAPK14 | mitogen-activated_protein_kinase_14 |
| ATP5A1 | ATP_synthase,_H+_transporting,_mitochondrial_F1_complex,_alpha_subunit_1 |
|  | **Discriminating genes with expressions positively correlated to CPT’s SOM NCI60 GI50 profile** |
| RAB13 | RAB13,_member_RAS_oncogene_family |
| NMT1 | N-myristoyltransferase_1 |
| EIF4A1 | eukaryotic_translation_initiation_factor_4A,_isoform_1 |
| SNRPG | small_nuclear_ribonucleoprotein_polypeptide_G |
| FEZ2 | fasciculation_and_elongation_protein_zeta_2_(zygin_II) |
| KHDRBS1 | KH_domain_containing,_RNA_binding,_signal_transduction_associated_1 |
| ACLY | ATP_citrate_lyase |
| **PSMD7** | proteasome_(prosome,_macropain)_26S_subunit,_non-ATPase |
| LSM6 | LSM6_homolog,_U6_small_nuclear_RNA_associated_(S._cerevisiae) |
| **PSMC1** | proteasome_(prosome,_macropain)_26S_subunit,_ATPase,_1 |
| EIF2S1 | eukaryotic_translation_initiation_factor_2,_subunit_1_alpha,_35kDa |
| **PSMB7** | proteasome_(prosome,_macropain)_subunit,_beta_type,_7 |
| **PSMA2** | proteasome_(prosome,_macropain)_subunit,_alpha_type,_2 |
| TERF1 | telomeric_repeat_binding_factor_(NIMA-interacting)_1 |
| EPB41L2 | erythrocyte_membrane_protein_band_4.1-like_2 |
| RPA3 | replication_protein_A3,_14kDa |
| **PSMC3** | proteasome_(prosome,_macropain)_26S_subunit,_ATPase,_3 |
| **PSMB3** | proteasome_(prosome,_macropain)_subunit,_beta_type,_3 |
| **PSMD12** | proteasome_(prosome,_macropain)_26S_subunit,_non-ATPase |
| TNFRSF1A | tumor_necrosis_factor_receptor_superfamily,_member_1A |
| MAP2K4 | mitogen-activated_protein_kinase_kinase_4 |
| HRAS | v-Ha-ras_Harvey_rat_sarcoma_viral_oncogene_homolog |
| RAD51C | RAD51_homolog_C_(S._cerevisiae) |
| ATRX | alpha_thalassemia/mental_retardation_syndrome_X-linked_(RAD54_homolog) |
| **PSMA4** | proteasome_(prosome,_macropain)_subunit,_alpha_type,_4 |
| CENPA | centromere_protein_A |
| HPRT1 | Hypoxanthine_phosphoribosyltransferase_1_(Lesch-Nyhan_syndrome) |
| KPNB1 | karyopherin_(importin)_beta_1 |
| **PSMD11** | proteasome_(prosome,_macropain)_26S_subunit,_non-ATPase,_11 |
| PRPS1 | phosphoribosyl_pyrophosphate_synthetase_1 |
| **PSMD9** | proteasome_(prosome,_macropain)_26S_subunit,_non-ATPase,_9 |
| TYMS | thymidylate_synthetase |
| PARD3 | Par-3_partitioning_defective_3_homolog_(C._elegans) |
| SNRPF | small_nuclear_ribonucleoprotein_polypeptide_F |
| FANCG | Fanconi_anemia,_complementation_group_G |
| CBX1 | chromobox_homolog_1_(HP1_beta_homolog_Drosophila_) |
| TRADD | TNFRSF1A-associated_via_death_domain |
| **PSMB6** | Proteasome_(prosome,_macropain)_subunit,_beta_type,_6 |
