# Supplementary material for: Integrating Constitutive Gene Expression and Chemoactivity: Mining the NCI60 Anticancer Screen
Source: PLoS One. 2012 Oct 2;7(10):e44631. doi: 10.1371/journal.pone.0044631 (PMC3462800; doi:10.1371/journal.pone.0044631)
Supplement: Table S2 — GSEA Results for Supplemental Test Compounds. (DOC) [file pone.0044631.s002.doc]

**Supplementary Table S2: GSEA Results for Supplemental Test Compounds:**

| **Geneset name** | **description** | **# genes in overlap (k)** | **p value** |  |
| --- | --- | --- | --- | --- |
|  |  |  |  |  |
| **Mitomycin C** |  |  |  |  |
| Kegg proteasome | Proteasome | 8 | 1.29E-10 |  |
| Biocarta proteasome pathway | Proteasome Complex | 5 | 4.07E-08 |  |
|  |  |  |  |  |
| **Digitoxin/Ouabain** |  |  |  |  |
| Kegg galactose metabolism | Galactose metabolism | 5 | 1.57E-06 |  |
| Carbohydrate kinase activity | GO:0019200. | 4 | 4.64E-06 |  |
|  |  |  |  |  |
| **Daunorubicin/Actinomysin** |  |  |  |  |
| DNA metabolic process | GO:0006259.. | 14 | 3.09E-12 |  |
| Nucleotide and nucleic acid metabolic process | GO:0006139. | 21 | 1.24E-08 |  |
| Kegg homologous recombination | Homologous recombination | 5 | 1.43E-07 |  |
| DNA repair | GO:0006281 | 7 | 1.22E-06 |  |
| DNA recombination | GO:0006310. | 5 | 2.10E-06 |  |
|  |  |  |  |  |
| **Dexamethasone** |  |  |  |  |
| DNA dependent ATPase activity | GO:0008094.. | 3 | 2.15E-05 |  |
| ATPase activity coupled | GO:0042623. | 4 | 7.74E-05 |  |
| Chromosomal part | GO:0044427 | 4 | 9.14E-05 |  |
| ATPase activity | GO:0016887. | 4 | 1.50E-04 |  |
|  |  |  |  |  |
| **Rapamycin** |  |  |  |  |
| Mitochondrion | GO:0005739 | 24 | 7.98E-14 |  |
| Cytoplasmic part | GO:0044444 | 44 | 9.96E-13 |  |
| Mitochondrial inner membrane | GO:0005743 | 11 | 9.20E-11 |  |
| Organelle inner membrane | GO:0019866 | 11 | 3.27E-10 |  |
| Organelle membrane | GO:0031090. | 18 | 1.36E-09 |  |
| Mitochondrial membrane | GO:0031966 | 11 | 1.48E-09 |  |
| Kegg oxidative phosphorylation | Oxidative phosphorylation | 13 | 1.59E-09 |  |
| Mitochondrial part | GO:0044429. | 13 | 2.73E-09 |  |
| Mitochondrial envelope | GO:0005740 | 11 | 4.89E-09 |  |
| Aerobic respiration | GO:0009060. | 6 | 5.86E-09 |  |
|  |  |  |  |  |
| **Vorinostat** |  |  |  |  |
| Calcium ino transmembrane transporter activity | GO:0015085. | 3 | 2.49E-05 |  |
| Cation transporting ATPase activity | GO:0019829. | 3 | 2.49E-05 |  |
| Cytoplasmic part | GO:0044444. | 19 | 5.64E-05 |  |
| Intracellular transport | GO:0046907. | 8 | 1.02E-04 |  |
| ATPase activity coupled transmembrane movement of ions phosphorylative mechanism | GO:0015662. | 3 | 1.66E-04 |  |
| Golgi appartus part | GO:0044431.. | 5 | 1.89E-04 |  |
|  |  |  |  |  |
| **Bortezomib** |  |  |  |  |
| Response to oxidative stress | GO:0006979 | 4 | 5.86E-05 |  |
| Transferase activity transferring phosphorous containing groups | GO:0016772 | 9 | 1.20E-04 |  |
| Phosphotransferase activity alcohol group as acceptor | GO:0016773. | 8 | 1.31E-04 |  |
|  |  |  |  |  |
| **Sangivamycin** |  |  |  |  |
| Translation | GO:0006412.. | 24 | 0.00E+00 |  |
| Structural molecule activity | GO:0005198. | 35 | 0.00E+00 |  |
| RNA binding | GO:0003723.. | 37 | 0.00E+00 |  |
| Structural constituent of ribosome | GO:0003735.. | 34 | 0.00E+00 |  |
| Kegg ribosome | Ribosome | 39 | 0.00E+00 |  |
|  |  |  |  |  |
| **Lovastatin/Simvastatin** |  |  |  |  |
| Kegg oxidative phosphorylation | Oxidative phosphorylation | 9 | 5.63E-06 |  |
| Kegg parkinsons disease | Parkinson's disease | 8 | 4.04E-05 |  |
| Kegg Hunington’s disease | Huntington's disease | 9 | 6.97E-05 |  |
| Cytoplasm | GO:0005737 | 36 | 1.10E-04 |  |
| Cytoskeleton | GO:0005856. | 12 | 1.71E-04 |  |
| Kegg Alzheimer’s disease | Alzheimer's disease | 8 | 2.17E-04 |  |
|  |  |  |  |  |
| **Cephalostatin/Schweinfurthin** |  |  |  |  |
| Oxidoreductase activity | GO:0016706. | 3 | 3.30E-05 |  |
| Oxidoreductase activity | GO:0016491. | 10 | 4.33E-05 |  |
|  |  |  |  |  |
| **Adaphostin** |  |  |  |  |
| Biocarta Tcr pathway | T Cell Receptor Signaling Pathway | 6 | 3.48E-07 |  |
| Biocarta Pyk2 pathway | Links between Pyk2 and Map Kinases | 5 | 7.49E-07 |  |
| Kegg neurotrophin signalling pathway | Neurotrophin signaling pathway | 8 | 8.36E-07 |  |
|  |  |  |  |  |
| **Thiosemicarbazone** |  |  |  |  |
| Kegg oxidative phosphorylation | Oxidative phosphorylation | 21 | 1.33E-15 |  |
| Kegg Parkinson’s disease | Parkinson's disease | 17 | 2.11E-11 |  |
| Kegg Huntington’s disease | Huntington's disease | 19 | 6.48E-11 |  |
| Kegg ribosome | Ribosome | 14 | 6.77E-11 |  |
| RNA binding | GO:0003723. | 21 | 1.16E-10 |  |
| Kegg Alzheimer’s disease | Alzheimer's disease | 18 | 1.16E-10 |  |
|  |  |  |  |  |
| **Acetogenins** |  |  |  |  |
| Mitochondrial inner membrane | GO:0005743. | 9 | 1.97E-11 |  |
| Mitochondrion | GO:0005739. | 15 | 2.27E-11 |  |
| Organelle inner membrane | GO:0019866 | 9 | 5.64E-11 |  |
| Mitochondrial membrane | GO:0031966.. | 9 | 1.99E-10 |  |
|  |  |  |  |  |
| **Parthenolide** |  |  |  |  |
| Proteasome complex | GO:0000502 | 6 | 3.31E-08 |  |
| Kegg proteasome | Proteasome | 7 | 1.66E-07 |  |
| Biocarta MAPK pathway | MAPKinase Signaling Pathway | 7 | 9.99E-06 |  |
