# Supplementary material for: Integrating Constitutive Gene Expression and Chemoactivity: Mining the NCI60 Anticancer Screen
Source: PLoS One. 2012 Oct 2;7(10):e44631. doi: 10.1371/journal.pone.0044631 (PMC3462800; doi:10.1371/journal.pone.0044631)
Supplement: Text S1 — The Supplementary Test Compounds section in Text S1 and Table S2, includes results for an additional 18 test compounds, the GSEA pathways identified by their discriminating gene and supporting literature validations. In principal, each SOM node can yield a set of discriminating genes that can be associated with its cluster members. Selecting an appropriate SOM node will depend, in part, on special interest in a set of compounds or a set of discriminating genes. (DOC) [file pone.0044631.s003.doc]

**Supplementary Material:**

**Supplemental Table S1**. U133A-derived genes selected to discriminate sensitive versus insensitive tumor cell responses to CPT. Genes are ordered from top to bottom according to correlation strength. Top 16 genes represent discriminating genes with expressions negatively correlated to CPT’s SOM NCI60 GI50 profile, where over expression corresponds to chemo-insensitivity. The bottom 38 discriminating genes have expressions positively correlated to CPT’s SOM NCI60 GI50 profile, with over expression corresponding to CPT chemo-sensitivity. Proteasomal genes are highlighted in bold.

**Supplementary Test Compounds.** The results for an additional eighteen test compounds (mitomycin C, digitoxin, ouabain, daunorubicin, actinomycin, dexamethasone. rapamycin, bortezimib, sangivamycin, vorinostat, lovastatin/simvastatin, cephalostatin/schweinfurthin, adaphostin, thiosemicarbazone, acetogenins and parthenolide) are described below. The GSEA results for these compounds appear collectively in **Supplementary Table S2**, labeled **GSEA Results for Supplemental Test Compounds.** These results are based on the U133A dataset.

The **mitomycin C** SOM cluster also appears as a CPT neighbor. The top scoring GSEA pathways are, as with CPT, the KEGG and Biocarta proteasome pathways. Consistent with the appearance of proteasome pathways in mitomycin C sensitivity, literature validation finds that DNA adducts of mytomycin effectively kill tumor cells as a result of proteasome-mediated degradation of checkpoint protein 1 (CHK1) [^1^](#_ENREF_1). Thus both mitomycin C and CPT yield similar GSEA pathways, yet the role of the proteasome appears to manifests itself differently for these two compounds by degrading different cellular products, both results consistent with literature reports.

**Digitoxin** and **ouabain** appear in the same SOM cluster, also near the CPT cluster. The top scoring GSEA pathways involve galactose metabolism and carbohydrate metabolism. While digitoxin and ouabain are well known to act as poisons of the Na-K-ATPase membrane pump[^2^](#_ENREF_2), validation of their role in sugar metabolism has been established in previous literature reports. Glucose and galactose are transported through a Na/glucose symporter in a sodium dependent mechanism, where the energy of transport derives from a Na-K ATPase membrane pump[^3^](#_ENREF_3). Movement of these moieties is facilitated by the glucose transporter isoform GLUT2[^4^](#_ENREF_4). Although the effect is indirect, the GI50 sensitivity profile and discriminating genes for digitoxin and ouabain provide sufficient information to suggest, via GSEA, a cellular effect of digitoxin and ouabain on carbohydrate metabolism that is consistent with literature sources.

**Daunorubicin** and **actinomycin D** appear in the same SOM GI50 cluster. The top-ranked GSEA pathways all involve DNA metabolism, either in chemical reactions involving DNA or DNA repair. Actinomycin D is an anti-tumor drug that intercalates into transcriptionally active regions of chromosomal DNA and thereby abrogates RNA synthesis of various transcriptional genes [^5^](#_ENREF_5). Daunorubicin produces DNA breaks mediated solely by Topoisomerase 2 (Topo2)[^6^](#_ENREF_6). In this case Topo2a has the highest positive correlation with GI50 sensitivity of its 47 discriminating genes, while MRE11A expression has the largest negative correlation with GI50 sensitivity. Here, the over expression of a known target (Topo2a) appears to correlate well with cellular sensitivity. Correspondingly, the role of over expressed MRE11A in drug insensitivity finds literature validation in the PKC-mediated recruitment of the complex Rad50/MRE11/NBS1 to repair damaged DNA[^7^](#_ENREF_7).

**Dexamethasone** is a potent member of the glucocorticoid class of steroid drugs that significantly increases Na^+^/K^+^ ATPase activity[^8^](#_ENREF_8). The GSEA results for dexamethasone strongly support this role. A feature unique to this result is that over expression of discriminating genes in the ATPase pathway corresponds to dexamethasone insensitivity. Chen et al [^9^](#_ENREF_9) find dexamethasone to enhance resistance to chemotherapy by increasing adhesion to extracellular matrix in human ovarian cancer cells. In addition, the NCI60 ovarian cell line SK-OV-3 exhibits a unique dexamethasone sensitivity to the over expression of ID1 (inhibitor of differentiation or DNA binding protein 1), among its discriminating genes, and the under expression of many ATPase pathway genes. ID1 contributes to tumorigenesis by stimulating cell proliferation, inhibiting cell differentiation and facilitating tumor neoangiogenesis [^10^](#_ENREF_10). Elevated ID1 is found in ovarian cancers [^11^](#_ENREF_11) and its level correlates with the malignant potential of ovarian tumors. Therefore, ID1 is a potential target for ovarian cancer treatment[^12^](#_ENREF_12). These results suggest a role for dexamethasone treatment of ovarian tumors having gene expression profiles similar to that of SK-OV-3.

FRAP, also known as mTOR or RAFT, is a FKBP12-**rapamycin** associated protein. FRAP is a member of the phosphatidylinositol kinase-related kinase family involved in signals that regulate cell growth in response to nutrients, cAMP levels and osmotic stress to control cellular transcription, translation, and autophagy[^13^](#_ENREF_13). FRAP has recently been reported to be regulated by mitochondrial dysfunction, where exposure to hyperosmotic conditions results in rapid and reversible dissipation of the mitochondrial proton gradient[^14^](#_ENREF_14). Mitochondrial dysfunction is manifested by FRAP’s association with the mitochondrial outer membrane. The top scoring rapamycin GSEA results implicate mitochondrial/organelle membranes in 5 of the top 6 scoring GSEA pathways. Consistent with literature reports, six of its discriminating genes appear in the aerobic metabolism pathway (GO:0009060)[^13^](#_ENREF_13), thus supporting rapamycin’s postulated role in autophagy[^15^](#_ENREF_15).

**Bortezomib** (Velcade) is a potent and reversible proteosome inhibitor with activity against many tumor types. Inhibition of the 26S proteosome induces apoptosis that is blocked by Bcl-2, an inhibitor of oxidative stress and mitochondrial injury[^16^](#_ENREF_16). Bortezomib’s GSEA results clearly support the role of oxidative phosphorylation as a means to generate reactive oxygen species (ROS) and apoptosis induction. Examination of bortezomib’s discriminating genes finds that over expression of glutathione synthetase (GSS), glutamate-cysteine ligase catalytic subunit (GCLC) and glutamate-cysteine ligase modifier subunit (GCLM) correlate with tumor cell sensitivity [^17^](#_ENREF_17). Interestingly, no proteasome pathways appear in bortezemib’s the top-scoring GSEA hits. Inspection of the NCI60 data finds that proteasome gene’s expressions do not correlate strongly with bortezemib’s SOM GI50 profiles. Here the stronger correlation is found with genes associated with the cellular response to oxidative stress (GSS, GCLC and GCLM).

**Sangivamycin** is a pro-apoptotic nucleoside, isolated from Streptomyces, with inhibitory effects on RNA/DNA/protein synthesis, protein kinase C and transcription elongation[^18^](#_ENREF_18). The over expression of 41 ribosomal proteins involved in translation and RNA binding are correlated with sangivamycin sensitivity, consistent with as its GSEA results implicating the ribosome and ribosomal function. Interestingly, GSEA over expressed discriminating genes that correlate with sangivamycin insensitivity are associated with non-membrane nuclear components and the cellular scaffolding components of the cytoskeleton. Apparently over expression of these genes provides a measure of cellular protection against the effects of sangivamycin on ribosomal-related cellular functions.

**Vorinostat** is a known HDAC inhibitor[^19^](#_ENREF_19). Its GSEA pathways reflect calcium transport as important for chemoactivity. Over expressed transport genes COG2 (component of oligomeric golgi complex 2), LRPPRC (leucine-rich PPR-motif containing), SDHC (succinate dehydrogenase complex, subunit C, integral membrane protein, 15kDa), DUT (dUTP pyrophosphatase) and ACY1 (aminoacylase 1) are positively correlated with GI50 chemoactivity. Literature evidence finds that defects of mitochondrial function and Ca^2+^ handling can be reduced with the administration of histone deacetylase (HDAC) inhibitors, effects believed to be related to more efficient Ca^2+^ handling[^20^](#_ENREF_20). Recently HDAC inhibitors have also been found to regulate claudin-1 expression in colon cancer cells [^21^](#_ENREF_23). Claudins function in establishing tight junctions needed to maintain cell-cell adhesion and regulate cellular permeability necessary for cell polarity. Agents that disrupt their normal membrane functions, such as calcium transport, would lead to defects manifest in the GSEA pathways listed here for vorinostat.

**Lovastatin** and **simvastatin** appear within the same SOM cluster. These cholesterol- lowering agents are hydroxymethylglutaryl coenzyme A (HMG-CoA) reductase inhibitors. HMG-CoA reductase inhibitors block 3-hydroxy-3-methylglutaryl coenzyme A reductase, the rate-limiting enzyme in cholesterol synthesis[^22^](#_ENREF_24). Consistent with oxidative phosphorylation as its top scoring GSEA, simvastatin has been found to inhibit mitochondrial respiratory Complex I, II and III systems [^23^](#_ENREF_25). The appearance of Parkinson’s, Huntington’s and Alzheimer’s diseases in its top scoring GSEA pathways is most likely due to the role of oxidative phosphorylation in these diseases[^24^](#_ENREF_26). The occurrence of simvastatin-induced myopathy in patients suggests that interference with the mitochondrial respiratory chain may also play a role in the toxicity of this class of drugs. Interestingly, all of its 9 discriminating genes within the mitochondrial oxidative phosphorylation pathway are inversely correlated with GI50 sensitivity. This observation may suggest that toxicity is enhanced for cells which under express these respiratory genes and diminished in cells where they are over expressed. In other words, cells with lowered mitochondrial respiration gene expressions may be more vulnerable to simvastatin-induced mitochondrial injury[^23^](#_ENREF_25).

**Cephalostatin** is a marine natural product with human tumor cell cytotoxicity. Rudy et al [^25^](#_ENREF_27)show that cephalostatin 1 induces apoptosis by using Smac/DIABLO (second mitochondria-derived activator of caspases/direct inhibitor of apoptosis-binding protein with a low isoelectric point) as a mitochondrial signaling molecule. Additional studies using ischemia-induced oxidative injury found increased formation of oxidized carbonyls in XIAP, mitochondrial Smac/DIABLO, and caspase-9[^26^](#_ENREF_28). Its GSEA pathways point strongly to oxidative stress as a component of cephalostatin’s cellular activity. However the initial trigger producing this oxidative stress remains unknown. The over-expressed GI50 sensitive genes include P4HA1 (procollagen-proline, 2-oxoglutarate 4-dioxygenase (proline 4-hydroxylase), alpha polypeptide I), P4HA2 (procollagen-proline, 2-oxoglutarate 4-dioxygenase (proline 4-hydroxylase), alpha polypeptide II) and PLOD3 (procollagen-lysine, 2-oxoglutarate 5-dioxygenase 3). All of these genes participate in the formation of collagen. **Schweinfurthins**, prenylated stilbene natural products isolated from a plant native to Cameroon[^27^](#_ENREF_29), appear in the same SOM node as cephalostatins. Interestingly the growth inhibitory effects of schweinfurthin A suggest selective activity against Neurofibromatosis type 1 (NF1) defective tumor cells[^28^](#_ENREF_30). The NF1 tumor suppressor regulates mouse skin wound healing, fibroblast proliferation, and collagen deposited by fibroblasts. NF1 mutant fibroblasts show abnormalities in collagen deposition in vitro [^29^](#_ENREF_31). Neurofibroma fibroblasts and Schwann cells are embedded in an extensive connective tissue matrix containing a large amount of collagens, proteoglycans, fibronectin, and laminin[^30^](#_ENREF_32) [^31^](#_ENREF_33). Up to 70% of the tumor dry weight of a neurofibroma is collagen[^32^](#_ENREF_34). Type I collagen is the major species, with collagens types III and V present in lesser amounts. Neurofibroma fibroblasts, which account for 20%–60% of the cells within the tumors, synthesize collagen I and III [^33^](#_ENREF_35) [^34^](#_ENREF_36). The GSEA results, combined with the over-expressed genes involved in collagen synthesis, suggest that schweinfurthin’s role in NF1 growth may involve interference in collagen formation.

The adenosine triphosphate binding-site-directed agent, STI571, and the tyrphostin, **adaphostin**, have been evaluated as bcr/abl kinase inhibitors[^35^](#_ENREF_37). These studies found that the differences in the activities of STI571 and adaphostin suggest that adaphostin is killing cells, at least in part, by a mechanism that does not involve the inhibition of p210^bcr/abl^-mediated signaling. Support for this observation comes from Losiewicz et al [^36^](#_ENREF_38) where AG957, a closely related analogue of adaphostin, is capable of inhibiting T-cell receptor-mediated c-Cbl phosphorylation in Jurkat cells. Because c-Cbl is a substrate of bcr/abl [^37^](#_ENREF_39), the observation that c-Cbl phosphorylation is inhibited in AG957-treated lymphoid cells raises the possibility that AG957 and its derivative, adaphostin, are exerting their effects by inhibiting other kinases that phosphorylate c-Cbl and p210^bcr/abl^. The finding that pathways for T-cell receptor signaling and neurotrophin signaling appear as the 1^st^ and 3^rd^ GSEA pathways for adaphostin support the possibility of a non-bcr/abl target. While recent findings for oxidative phosphorylation as a mechanism of action for adaphostins would be consistent with the SOM GI50 neighbor of acetogenins, sufficient information regarding the gene expression profile supports T-cell receptor-mediated signaling, and possible neurotoxicity as important considerations for adaphostin therapy.

Considerable interest has been directed at the use of metal chelating agents in anticancer therapy[^38^](#_ENREF_40). Early examination of a series of substituted bis(**thiosemicarbazone**) copper(II) complexes finds their reaction with tumor cells to involve complexes with sulfhydryl groups, where reactivity correlates directly with the ability to inhibit DNA synthesis and cellular respiration[^39^](#_ENREF_41). While the GSEA pathway results clearly point to the importance of oxidative phosphorylation in thiosemicarbazone’s cellular activity, recent studies have examined further the role of reactive oxygen species in tumor cell killing. Specifically they observed that thiosemicarbazone is most sensitive in cells with down-regulation of P-glycoprotein (P-gp); leading to the conclusion that loss of P-gp results in sensitization to drugs that are P-gp substrates. GSEA pathway analysis of the subset of thiosemicarbazone discriminating genes having only a negative correlation with GI50 sensitivity identifies Golgi apparatus (GO:0005794 p<2.16e-5) and endoplasmic reticulum (GO:0005793 p<1.0e-4) as the top scoring GSEA pathways. Fluorescence microscopic observations showed that the P-gp drug resistance transporter, in addition to being located at the cell surface, can also be found in the Golgi apparatus of resistant cells[^40^](#_ENREF_42). Seven of the inversely correlated thiosemicarbazone discriminating genes (EXT1; exostoses (multiple) 1, EXT2; exostoses (multiple) 2, ACO1; aconitase 1, soluble, CAV1; caveolin 1, ORCL; oculocerebrorenal syndrome of Lowe, IGF2R; insulin-like growth factor 2 receptor and OPTN; optineurin) appear in the GO:0005794, Golgi appartus pathway. These results support the findings that thiosemicarbazone is most sensitive in cells under expressing these Golgi-specific enzymes. Discriminating genes EXT1/2 are tumor suppressors where germline mutations and functional loss are commonly found in multiple osteochondromas and predispose to the development of chondrosarcoma[^41^](#_ENREF_43). The gene products, exostosin-1 (EXT1) and exostosin-2 (EXT2), are type II transmembrane glycoproteins which form Golgi-localized complexes that catalyze polymerization of heparan sulfate (HS) [^42^](#_ENREF_44). Components of the extracellular environment direct cell proliferation, differentiation, migration, and changes in cell shape by binding to highly specific cell surface receptors, which, when occupied, are either internalized or transduce intracellular signals. These receptors transmit signals into the cell and often use cell surface heparan sulfate (HS) to recognize their ligands or to regulate their activation. HS is synthesized on a variety of cell surface proteins but is found consistently on families of membrane-bound proteoglycans (PGs), the syndecans and the glypicans[^43^](#_ENREF_45). Together these results are consistent with thiozemicarbazone as a P-gp substrate and the existence of glycoprotein up regulation being associated with cellular insensitivity.

Acetogenins, such as **bullatacin**, **asimicin** and **rollitacin**, are isolated from plants of the Annonaceae, and show potent activity in the inhibition of murine tumor growth and human tumor xenografts[^44^](#_ENREF_46) through depletion of ATP levels via inhibiting complex I of mitochondria[^45^](#_ENREF_47) and inhibiting the NADH oxidase of plasma membranes of tumor cells. These literature-reported activities are consistent with the identification of mitochondria and mitochondrial-related functions as acetogenins’ top scoring GSEA pathways. Inspection of the most over expressed discriminating genes that positively correlate with GI50 sensitivity finds; UQCRB (ubiquinol-cytochrome c reductase binding protein), ATP5D (ATP synthase, H+ transporting, mitochondrial F1 complex, delta subunit), COX7A2L (cytochrome c oxidase subunit VIIa polypeptide 2 like) and ACO2 (aconitase 2, mitochondrial). These results are consistent with acetogenins affecting mitochondrial function. **Oligomycin**, also found in this same SOM GI50 cluster, is a highly specific mitochondrial ATP-synthase inhibitor[^46^](#_ENREF_48).

**Parthenolide**, a sesquiterpene lactone found in several plants including Feverfew and Magnolia[^47^](#_ENREF_49), acts to deplete histone deacetylase [^48^](#_ENREF_50). A critical role of histone deacetylase 1 (HDAC1) in the development of cancer is through interactions with transcription factors. Thus inhibition of the HDAC1 enzyme can control cancer cell growth and development. Parthenolide activates the ATM enzyme, which results in the depletion of the histone deacetylase 1 (HDAC1) from the cells and cell death. The apoptotic signals triggered by DNA damage also activate tumor suppressors such as p53 to promote synthesis of p21 and p27, which restrict cell cycle progression through both the G1 and G2 portions of the cell cycle[^48^](#_ENREF_50). HDAC1 is antagonistic to p53 and prevents this cell cycle inhibitor mediated cellular death. Parthenolide directly or indirectly modifies the HDAC1 enzyme, which can restore the expressions of some suppressor genes such as p53. This modification results in proteasomal degradation of HDAC1. Parthenolide may overcome the resistance to chemotherapeutic drugs by restoring the p53 tumor-repressor function via its hyper-acetylation and nuclear migration, events usually impaired in tumors. Inhibition of NF-kB activity, constitutive in many types of cancers, is also considered one of the main mechanisms of parthenolide’s action. The GSEA analysis of parthenolide clearly supports the importance of the proteasome in its tumor cell sensitivity. The GSEA results, however, also support the importance of NF-kb activity in tumor cell sensitivity. Regulation of NF-kB activity apparently occurs through p38 MAP kinase-mediated modulation of CBP, an NF-kB coactivator protein. p38 MAP kinase regulates the transcriptional activation function of the p65 subunit of NF-kB without directly targeting NF-kB. Instead, p38 can interact with and phosphorylate the CBP an essential coactivator for NF-kB. Thus inhibition of p38 kinase activity reduces the amount of CBP that interacts with the p65 subunit of NF-kB; an observation supporting its GSEA p38 kinase activity in parthenolide’s chemoactivity.

**Supplementary Table S2: GSEA Results for Supplemental Test Compounds:**

**References**

1. Boamah, E. K.; Brekman, A.; Tomasz, M.; Myeku, N.; Figueiredo-Pereira, M.; Hunter, S.; Meyer, J.; Bhosle, R. C.; Bargonetti, J., DNA adducts of decarbamoyl mitomycin C efficiently kill cells without wild-type p53 resulting from proteasome-mediated degradation of checkpoint protein 1. *Chem Res Toxicol* **2010,** *23* (7), 1151-62.

2. Newman, R. A.; Yang, P.; Pawlus, A. D.; Block, K. I., Cardiac glycosides as novel cancer therapeutic agents. *Mol Interv* **2008,** *8* (1), 36-49.

3. Thorens, B., Facilitated glucose transporters in epithelial cells. *Annu Rev Physiol* **1993,** *55*, 591-608.

4. Yasuda, K.; Yamada, Y.; Inagaki, N.; Yano, H.; Okamoto, Y.; Tsuji, K.; Fukumoto, H.; Imura, H.; Seino, S.; Seino, Y., Expression of GLUT1 and GLUT2 glucose transporter isoforms in rat islets of Langerhans and their regulation by glucose. *Diabetes* **1992,** *41* (1), 76-81.

5. Mischo, H. E.; Hemmerich, P.; Grosse, F.; Zhang, S., Actinomycin D induces histone gamma-H2AX foci and complex formation of gamma-H2AX with Ku70 and nuclear DNA helicase II. *The Journal of biological chemistry* **2005,** *280* (10), 9586-94.

6. Ciesielska, E.; Studzian, K.; Wasowska, M.; Oszczapowicz, I.; Szmigiero, L., Cytotoxicity, cellular uptake and DNA damage by daunorubicin and its new analogues with modified daunosamine moiety. *Cell Biol Toxicol* **2005,** *21* (3-4), 139-47.

7. Assenmacher, N.; Hopfner, K. P., MRE11/RAD50/NBS1: complex activities. *Chromosoma* **2004,** *113* (4), 157-66.

8. Ullrich, S.; Zhang, Y.; Avram, D.; Ranta, F.; Kuhl, D.; Haring, H. U.; Lang, F., Dexamethasone increases Na+/K+ ATPase activity in insulin secreting cells through SGK1. *Biochemical and biophysical research communications* **2007,** *352* (3), 662-7.

9. Chen, Y. X.; Wang, Y.; Fu, C. C.; Diao, F.; Song, L. N.; Li, Z. B.; Yang, R.; Lu, J., Dexamethasone enhances cell resistance to chemotherapy by increasing adhesion to extracellular matrix in human ovarian cancer cells. *Endocr Relat Cancer* **2010,** *17* (1), 39-50.

10. Takai, N.; Miyazaki, T.; Fujisawa, K.; Nasu, K.; Miyakawa, I., Id1 expression is associated with histological grade and invasive behavior in endometrial carcinoma. *Cancer Lett* **2001,** *165* (2), 185-93.

11. Li, Z. D.; Hu, X. W.; Wang, Y. T.; Fang, J., Apigenin inhibits proliferation of ovarian cancer A2780 cells through Id1. *FEBS letters* **2009,** *583* (12), 1999-2003.

12. Coppe, J. P.; Smith, A. P.; Desprez, P. Y., Id proteins in epithelial cells. *Exp Cell Res* **2003,** *285* (1), 131-45.

13. Desai, B. N.; Myers, B. R.; Schreiber, S. L., FKBP12-rapamycin-associated protein associates with mitochondria and senses osmotic stress via mitochondrial dysfunction. *Proceedings of the National Academy of Sciences of the United States of America* **2002,** *99* (7), 4319-24.

14. Goldenthal, M. J.; Marin-Garcia, J., Mitochondrial signaling pathways: a receiver/integrator organelle. *Mol Cell Biochem* **2004,** *262* (1-2), 1-16.

15. Raught, B.; Gingras, A. C.; Sonenberg, N., The target of rapamycin (TOR) proteins. *Proceedings of the National Academy of Sciences of the United States of America* **2001,** *98* (13), 7037-44.

16. Dasmahapatra, G.; Lembersky, D.; Rahmani, M.; Kramer, L.; Friedberg, J.; Fisher, R. I.; Dent, P.; Grant, S., Bcl-2 antagonists interact synergistically with bortezomib in DLBCL cells in association with JNK activation and induction of ER stress. *Cancer biology & therapy* **2009,** *8* (9), 808-19.

17. Du, Z. X.; Zhang, H. Y.; Meng, X.; Guan, Y.; Wang, H. Q., Role of oxidative stress and intracellular glutathione in the sensitivity to apoptosis induced by proteasome inhibitor in thyroid cancer cells. *BMC cancer* **2009,** *9*, 56.

18. Stockwin, L. H.; Yu, S. X.; Stotler, H.; Hollingshead, M. G.; Newton, D. L., ARC (NSC 188491) has identical activity to Sangivamycin (NSC 65346) including inhibition of both P-TEFb and PKC. *BMC cancer* **2009,** *9*, 63.

19. Hrzenjak, A.; Moinfar, F.; Kremser, M. L.; Strohmeier, B.; Petru, E.; Zatloukal, K.; Denk, H., Histone deacetylase inhibitor vorinostat suppresses the growth of uterine sarcomas in vitro and in vivo. *Mol Cancer* **2010,** *9*, 49.

20. (a) Krishnan, M.; Singh, A. B.; Smith, J. J.; Sharma, A.; Chen, X.; Eschrich, S.; Yeatman, T. J.; Beauchamp, R. D.; Dhawan, P., HDAC inhibitors regulate claudin-1 expression in colon cancer cells through modulation of mRNA stability. *Oncogene* **2009,** *29* (2), 305-12; (b) Singh, A. B.; Sharma, A.; Dhawan, P., Claudin family of proteins and cancer: an overview. *J Oncol* **2010,** *2010*, 541957; (c) Oliveira, J. M.; Chen, S.; Almeida, S.; Riley, R.; Goncalves, J.; Oliveira, C. R.; Hayden, M. R.; Nicholls, D. G.; Ellerby, L. M.; Rego, A. C., Mitochondrial-dependent Ca2+ handling in Huntington's disease striatal cells: effect of histone deacetylase inhibitors. *J Neurosci* **2006,** *26* (43), 11174-86.

21. Krishnan, M.; Singh, A. B.; Smith, J. J.; Sharma, A.; Chen, X.; Eschrich, S.; Yeatman, T. J.; Beauchamp, R. D.; Dhawan, P., HDAC inhibitors regulate claudin-1 expression in colon cancer cells through modulation of mRNA stability. *Oncogene 29* (2), 305-12.

22. Wong, W. W.; Dimitroulakos, J.; Minden, M. D.; Penn, L. Z., HMG-CoA reductase inhibitors and the malignant cell: the statin family of drugs as triggers of tumor-specific apoptosis. *Leukemia* **2002,** *16* (4), 508-19.

23. Nadanaciva, S.; Bernal, A.; Aggeler, R.; Capaldi, R.; Will, Y., Target identification of drug induced mitochondrial toxicity using immunocapture based OXPHOS activity assays. *Toxicol In Vitro* **2007,** *21* (5), 902-11.

24. Shoffner, J. M.; Watts, R. L.; Juncos, J. L.; Torroni, A.; Wallace, D. C., Mitochondrial oxidative phosphorylation defects in Parkinson's disease. *Annals of neurology* **1991,** *30* (3), 332-9.

25. Rudy, A.; Lopez-Anton, N.; Barth, N.; Pettit, G. R.; Dirsch, V. M.; Schulze-Osthoff, K.; Rehm, M.; Prehn, J. H.; Vogler, M.; Fulda, S.; Vollmar, A. M., Role of Smac in cephalostatin-induced cell death. *Cell Death Differ* **2008,** *15* (12), 1930-40.

26. Saito, A.; Hayashi, T.; Okuno, S.; Nishi, T.; Chan, P. H., Oxidative stress is associated with XIAP and Smac/DIABLO signaling pathways in mouse brains after transient focal cerebral ischemia. *Stroke; a journal of cerebral circulation* **2004,** *35* (6), 1443-8.

27. Beutler, J. A.; Shoemaker, R. H.; Johnson, T.; Boyd, M. R., Cytotoxic geranyl stilbenes from Macaranga schweinfurthii. *Journal of natural products* **1998,** *61* (12), 1509-12.

28. Turbyville, T. J.; Gursel, D. B.; Tuskan, R. G.; Walrath, J. C.; Lipschultz, C. A.; Lockett, S. J.; Wiemer, D. F.; Beutler, J. A.; Reilly, K. M., Schweinfurthin A selectively inhibits proliferation and Rho signaling in glioma and neurofibromatosis type 1 tumor cells in a NF1-GRD-dependent manner. *Molecular cancer therapeutics* **2010,** *9* (5), 1234-43.

29. Atit, R. P.; Crowe, M. J.; Greenhalgh, D. G.; Wenstrup, R. J.; Ratner, N., The Nf1 tumor suppressor regulates mouse skin wound healing, fibroblast proliferation, and collagen deposited by fibroblasts. *J Invest Dermatol* **1999,** *112* (6), 835-42.

30. Uitto, J.; Matsuoka, L. Y.; Chu, M. L.; Pihlajaniemi, T.; Prockop, D. J., Connective tissue biochemistry of neurofibromas. *Ann N Y Acad Sci* **1986,** *486*, 271-86.

31. Konomi, H.; Arima, M.; Tanaka, H.; Hayashi, T.; Ikeda, S., Increased deposition of types III and V collagen in neurofibroma tissue from patients with von Recklinghausen disease. *Brain Dev* **1989,** *11* (6), 378-83.

32. Peltonen, J.; Jaakkola, S.; Lebwohl, M.; Renvall, S.; Risteli, L.; Virtanen, I.; Uitto, J., Cellular differentiation and expression of matrix genes in type 1 neurofibromatosis. *Laboratory investigation; a journal of technical methods and pathology* **1988,** *59* (6), 760-71.

33. Jaakkola, S.; Peltonen, J.; Riccardi, V.; Chu, M. L.; Uitto, J., Type 1 neurofibromatosis: selective expression of extracellular matrix genes by Schwann cells, perineurial cells, and fibroblasts in mixed cultures. *The Journal of clinical investigation* **1989,** *84* (1), 253-61.

34. Sollberg, S.; Muona, P.; Lebwohl, M.; Peltonen, J.; Uitto, J., Presence of type I and VI collagen mRNAs in endothelial cells in cutaneous neurofibromas. *Laboratory investigation; a journal of technical methods and pathology* **1991,** *65* (2), 237-42.

35. Mow, B. M.; Chandra, J.; Svingen, P. A.; Hallgren, C. G.; Weisberg, E.; Kottke, T. J.; Narayanan, V. L.; Litzow, M. R.; Griffin, J. D.; Sausville, E. A.; Tefferi, A.; Kaufmann, S. H., Effects of the Bcr/abl kinase inhibitors STI571 and adaphostin (NSC 680410) on chronic myelogenous leukemia cells in vitro. *Blood* **2002,** *99* (2), 664-71.

36. Losiewicz, M. D.; Kaur, G.; Sausville, E. A., Different early effets of tyrphostin AG957 and geldanamycins on mitogen-activated protein kinase and p120cbl phosphorylation in anti CD-3-stimulated T-lymphoblasts. *Biochem Pharmacol* **1999,** *57* (3), 281-9.

37. Sattler, M.; Salgia, R.; Okuda, K.; Uemura, N.; Durstin, M. A.; Pisick, E.; Xu, G.; Li, J. L.; Prasad, K. V.; Griffin, J. D., The proto-oncogene product p120CBL and the adaptor proteins CRKL and c-CRK link c-ABL, p190BCR/ABL and p210BCR/ABL to the phosphatidylinositol-3' kinase pathway. *Oncogene* **1996,** *12* (4), 839-46.

38. Coats, E. A.; Milstein, S. R.; Pleiss, M. A.; Roesener, J. A., Comparative analysis of the cytotoxicity of substituted [phenylglyoxal bis(4-methyl-3-thiosemicarbazone)] copper(II) chelates. 2. Parabolic correlations and their implications for selective toxicity. *Journal of medicinal chemistry* **1978,** *21* (8), 804-9.

39. Minkel, D. T.; Saryan, L. A.; Petering, D. H., Structure-function correlations in the reaction of bis(thiosemicarbazonato) copper(II) complexes with Ehrlich ascites tumor cells. *Cancer research* **1978,** *38* (1), 124-9.

40. Molinari, A.; Cianfriglia, M.; Meschini, S.; Calcabrini, A.; Arancia, G., P-glycoprotein expression in the Golgi apparatus of multidrug-resistant cells. *Int J Cancer* **1994,** *59* (6), 789-95.

41. Tsuchiya, T.; Osanai, T.; Ogose, A.; Tamura, G.; Chano, T.; Kaneko, Y.; Ishikawa, A.; Orui, H.; Wada, T.; Ikeda, T.; Namba, M.; Takigawa, M.; Kawashima, H.; Hotta, T.; Tsuchiya, A.; Ogino, T.; Motoyama, T., Methylation status of EXT1 and EXT2 promoters and two mutations of EXT2 in chondrosarcoma. *Cancer Genet Cytogenet* **2005,** *158* (2), 148-55.

42. Bao, X.; Moseman, E. A.; Saito, H.; Petryanik, B.; Thiriot, A.; Hatakeyama, S.; Ito, Y.; Kawashima, H.; Yamaguchi, Y.; Lowe, J. B.; von Andrian, U. H.; Fukuda, M., Endothelial heparan sulfate controls chemokine presentation in recruitment of lymphocytes and dendritic cells to lymph nodes. *Immunity* **2010,** *33* (5), 817-29.

43. Bernfield, M.; Gotte, M.; Park, P. W.; Reizes, O.; Fitzgerald, M. L.; Lincecum, J.; Zako, M., Functions of cell surface heparan sulfate proteoglycans. *Annual review of biochemistry* **1999,** *68*, 729-77.

44. Alali, F. Q.; Liu, X. X.; McLaughlin, J. L., Annonaceous acetogenins: recent progress. *Journal of natural products* **1999,** *62* (3), 504-40.

45. Glover, C. J.; Rabow, A. A.; Isgor, Y. G.; Shoemaker, R. H.; Covell, D. G., Data mining of NCI's anticancer screening database reveals mitochondrial complex I inhibitors cytotoxic to leukemia cell lines. *Biochem Pharmacol* **2007,** *73* (3), 331-40.

46. Chiu, H. F.; Chih, T. T.; Hsian, Y. M.; Tseng, C. H.; Wu, M. J.; Wu, Y. C., Bullatacin, a potent antitumor Annonaceous acetogenin, induces apoptosis through a reduction of intracellular cAMP and cGMP levels in human hepatoma 2.2.15 cells. *Biochem Pharmacol* **2003,** *65* (3), 319-27.

47. Piela-Smith, T. H.; Liu, X., Feverfew extracts and the sesquiterpene lactone parthenolide inhibit intercellular adhesion molecule-1 expression in human synovial fibroblasts. *Cell Immunol* **2001,** *209* (2), 89-96.

48. Gopal, Y. N.; Arora, T. S.; Van Dyke, M. W., Parthenolide specifically depletes histone deacetylase 1 protein and induces cell death through ataxia telangiectasia mutated. *Chemistry & biology* **2007,** *14* (7), 813-23.
